# Cross-domain diversity effects: linking diatom species richness, intraspecific richness, and biomass production to host-associated bacterial diversity

**DOI:** 10.1093/ismeco/ycae046

**Published:** 2024-03-29

**Authors:** Marrit Jacob, Patrick K Thomas, Helge-Ansgar Giebel, Sara Billerbeck, Meinhard Simon, Maren Striebel, Leon Dlugosch

**Affiliations:** Institute for Chemistry and Biology of the Marine Environment (ICBM), School of Mathematics and Science, Carl von Ossietzky Universität Oldenburg, Ammerländer Heerstraße 114-118, Oldenburg 26129, Germany; Marine Chemistry, Department of Biology and Chemistry, University of Bremen, James-Watt-Straße 1 (BIOM), Bremen 28359, Germany; Institute for Chemistry and Biology of the Marine Environment (ICBM), School of Mathematics and Science, Carl von Ossietzky Universität Oldenburg, Ammerländer Heerstraße 114-118, Oldenburg 26129, Germany; Department of Aquatic Ecology, Swiss Federal Institute of Aquatic Science and Technology (EAWAG), Überlandstrasse 133, 8600 Dübendorf, Switzerland; Institute for Chemistry and Biology of the Marine Environment (ICBM), School of Mathematics and Science, Carl von Ossietzky Universität Oldenburg, Ammerländer Heerstraße 114-118, Oldenburg 26129, Germany; Institute for Chemistry and Biology of the Marine Environment (ICBM), School of Mathematics and Science, Carl von Ossietzky Universität Oldenburg, Ammerländer Heerstraße 114-118, Oldenburg 26129, Germany; Institute for Chemistry and Biology of the Marine Environment (ICBM), School of Mathematics and Science, Carl von Ossietzky Universität Oldenburg, Ammerländer Heerstraße 114-118, Oldenburg 26129, Germany; Institute for Chemistry and Biology of the Marine Environment (ICBM), School of Mathematics and Science, Carl von Ossietzky Universität Oldenburg, Ammerländer Heerstraße 114-118, Oldenburg 26129, Germany; Institute for Chemistry and Biology of the Marine Environment (ICBM), School of Mathematics and Science, Carl von Ossietzky Universität Oldenburg, Ammerländer Heerstraße 114-118, Oldenburg 26129, Germany

**Keywords:** biodiversity and ecosystem functioning, microbial ecology, host-microbiome interactions, functional diversity, symbiosis, holobiont, algae-bacteria interactions, phycosphere, marine microbiology, environmental microbiology

## Abstract

Interactions between bacteria and microalgae are important for the functioning of aquatic ecosystems, yet interactions based on the biodiversity of these two taxonomic domains have been scarcely studied. Specifically, it is unclear whether a positive biodiversity–productivity relationship in phytoplankton is largely facilitated by niche partitioning among the phytoplankton organisms themselves or whether associated bacterial communities play an additional role in modifying these diversity effects. Moreover, the effects of intraspecific diversity in phytoplankton communities on bacterial community diversity have not been tested. To address these points, we factorially manipulated both species and intraspecific richness of three diatoms to test the effects of diatom species/strain diversity on biomass production and bacterial diversity in algae–bacteria communities. The results show that diatom intraspecific diversity has significant positive effects on culture biomass and the diversity of the associated free-living bacterial community (0.2–3 μm size fraction), which are comparable in magnitude to species diversity effects. However, there were little to no effects of diatom diversity on host-associated bacterial diversity (>3 μm size fraction), or of bacterial diversity on biomass production. These results suggest a decoupling of bacterial diversity from the diatom diversity-productivity relationship and provide early insights regarding the relations between diversity across domains in aquatic ecosystems.

## Introduction

Biodiversity influences how ecosystems function globally, from aquatic to terrestrial systems, by buffering the impact of environmental variability and enhancing the efficiency of resource use within ecosystems [1–3]. Most of what we know about biodiversity and ecosystem functioning (BEF), however, is related to diversity in terms of distinct species within one ecological guild (e.g. primary producers). For example, the Cedar Creek grassland experiments sparked a wave of research into BEF, which amassed a great number of studies documenting the consequences of biodiversity loss and exploring how mechanisms like niche complementarity among plants influence diversity effects [1, 4–6]. However, further investigations revealed that the observed diversity effects are in fact largely mediated by the microbial communities in grasslands [7], because plant-associated microbes tend to enhance nutrient cycling back to plants in more diverse polycultures [8]. Conversely, microbes can reduce productivity in low-diversity systems due to a greater prevalence of pathogenesis [7]. Thus, diversity across levels of biological organization can interactively determine ecosystem processes like productivity and nutrient cycling. This illustrates how an integrated understanding of cross-domain diversity effects is vital to gain insight into links between biodiversity and ecosystem processes in nature.

Like cross-domain diversity effects, the effects of intraspecific diversity in biodiversity-ecosystem functioning relationships were also disregarded for a long time. Increasing evidence suggests that intraspecific diversity effects can be as important in magnitude as species effects [9, 10]. This is attributed to substantial intraspecific trait variation in many organisms, as functional traits are more directly relevant for linking individuals to their interactions with the environment than taxonomic affiliations per se [11, 12]. However, this progress in trait-based ecology risks disregarding cross-domain interactions, which may shape how intraspecific variation influences the environment. To date, there is little knowledge on how intraspecific diversity in one guild can interact across guilds to shape ecosystem functioning.

Linking these two knowledge gaps (i.e. on biodiversity effects across domains and the impact of intraspecific diversity) and addressing them in combination is an important step in gaining a better understanding of complex biodiversity-ecosystem functioning relationships. Moreover, most data on cross-domain diversity effects come from terrestrial grassland systems. Phytoplankton make up near half of global primary production, with diatoms alone accounting for roughly 40% of marine primary production [13, 14]; therefore, diatoms and their microbiomes are of particular importance. Losses in phytoplankton diversity have been linked with declines in carbon export [15] and lower productivity [16], but little knowledge exists of how phytoplankton and bacterial diversity collectively influence ecosystem processes. The microbiome of phytoplankton (i.e. the “phycosphere,” reviewed by Seymour *et al.* [17]) substantially alters the productivity and fitness of their host [18–20], which has major implications for the global carbon cycle [15]. Bacteria can improve host productivity by supplying essential vitamins like cobalamin (Vitamin B_12_), soluble iron, or other growth factors to their host [21–23]. However, bacteria–algae interactions can take many forms, ranging from mutualistic to competitive or pathogenic [24]. Microalgae interact both with bacteria directly attached to their surface (“attached community,” often assessed by 16S rRNA gene metabarcoding of particles >3 μm in size) and with bacteria more loosely associated with the vicinity of the host (“free-living community,” often assessed by 16S rRNA gene metabarcoding of the size fraction 0.2–3 μm). These two subcommunities are compositionally distinct and can exhibit different functional properties [25, 26]. The nature of cross-domain interactions between microalgae and bacteria can be highly species- or even genotype-specific; one bacterium can stimulate the growth of one algal strain and inhibit another [27, 28]. This host-specificity has important implications for diversity-functioning relationships in phytoplankton. It implies that bacterial symbionts can potentially determine the diversity-productivity relationship depending upon: (i) whether greater phytoplankton diversity leads to greater bacterial diversity and (ii) whether the prevalent form of interaction linked to greater bacterial diversity is positive or negative. Addressing these outstanding questions would enhance our understanding of how bacterial diversity mediates the important role that phytoplankton play in global primary production and geochemical cycling.

In this study, we explicitly test how phytoplankton species richness and intraspecific richness influence bacterial taxonomic and functional diversity and, in turn, how bacterial diversity is linked to phytoplankton community productivity. We accomplish this using an experimental system of three cosmopolitan diatom species, with three strain isolates per species, to factorially test the relative importance of species and intraspecific diversity in shaping the diatom community microbiome. Specifically, we test the following hypotheses: (H1) individual diatom strains have a distinct host-specific microbiome, (H2) diatom inter- and intraspecific richness have positive and equal effects on community productivity, (H3) diatom inter- and intraspecific richness have positive and equal effects on bacterial diversity, and (H4) greater bacterial diversity is correlated with greater community productivity.

## Materials and methods

### Strains and culture maintenance

We focused on the diatom species *Ditylum brightwellii*, *Rhizosolenia setigera*, and *Thalassionema nitzschioides*, and isolated three strains of each species. Specifically, one strain of each species was isolated from seawater collected offshore from each of the following locations: Borkum (open North Sea, 53°53′32″N, 6°28′22″E), Helgoland (open North Sea, 54°12′17″N, 7°58′01″E), and Kiel (coastal Baltic Sea, 54°24′22″N, 10°12′22″E) in November 2019. For strain identification, each culture was filtered onto a 3 μm Whatman Nuclepore® polycarbonate filter; DNA extraction, PCR, and sequencing of the 18S rRNA gene were then performed as described by [29] using 18S primers from [30]. The results are given in the supplementary material (Table S1 and Fig. S1) and show slight variations among the three isolates of each diatom species. The isolates were cultured in sterile *f*/2 medium [31] at 25 ppt salinity in polystyrol bottles at 150 μmol photons m^−2^ s^−1^ and a 16:8 h day:night cycle. They were cultured for 7 months at 12–16°C and were acclimated to 18°C in the weeks prior to the experiment. Diatom growth was tracked as *in vivo* chlorophyll fluorescence (excitation at 460 nm, emission at 685 nm) on a microplate reader (Biotek Synergy H1®) and recorded as relative fluorescence units (RFU). For the experimental medium, trace mineral concentration was halved and nutrient content was reduced to 64 μM NO_3_, 60 μM SiO_3_, and 4 μM PO_4_ to approximate the Redfield ratio in naturally occurring concentrations [32, 33]. A fresh seawater inoculum containing naturally occurring bacterial communities (sampled from Jade Bight, 53°31′05″N, 8°09′36″E) was filtered twice using a peristaltic pump (Cole-Palmer Masterflex L/S®; 9 mm diameter silicone tube at 15 rpm, 3 μm pore size) and added to the laboratory cultures to allow for the establishment of a refreshed bacterial community after several months of lab culturing. Diatom cultures in exponential growth were washed by gravity filtration (5 μm pore size) and resuspended in sterile-filtered seawater to equal densities in terms of *in vivo* fluorescence (55 RFU), with the bacterial inoculum making up 17% of the resuspension volume. The laboratory cultures without bacterial inoculum are hereafter referred to as “original cultures,” while the cultures that received the bacterial inoculum are referred to as “inoculated cultures.” All cultures used in the experiment were incubated at 18°C for 36 h.

### Experimental setup

Species and intraspecific strain diversity were factorially manipulated from the freshly inoculated cultures to create four diversity levels: 1 species/1 strain (monocultures), 1 species/3 strains (intraspecific polycultures), 3 species/1 strain of each species (species polycultures), and 3 species/3 strains of each species (full polyculture), which resulted in a total of 16 unique treatment combinations (see Table S2). Individual treatments were run in triplicates (some samples were lost, however; see Table S2). The initial biomass of the cultures was standardized to 55 RFU again after the initial inoculation phase of 36 h. Experimental units (1 L Duran® borosilicate bottles) were inoculated according to the assigned diversity treatment using equivalent RFU among the included strains, adding up to a cumulative value of 10 RFU, corresponding roughly to a biovolume of 6 × 10^6^ μm^3^ ml^−1^ (SD ± 2.9 × 10^6^). Original cultures were run as unreplicated controls for the inoculated cultures. The experimental units were incubated at 18°C under LED panel lamps (OUBO brand, 3000 K warm white spectrum) at 141 (SD ± 22) μmol photons m^−2^ s^−1^ and a 16:8 h day:night cycle. Their position was randomized daily. The mean RFU of two technical replicates (1.6 ml each) per experimental unit was used to track growth.

### Sampling

Samples for bacterial community analysis were taken in the late exponential/early stationary phase (see Fig. S2 for growth curves) by filtering 500 ml of culture using a peristaltic pump (Masterflex L/S®, Cole-Palmer, 9 mm diameter silicone tubing, 10 rpm) in series over a 3 μm pore size filter (Whatman Nuclepore®) to collect the host surface-attached community, followed by a 0.2 μm pore size filter (Whatman Cyclopore®) to collect the free-living community. Filters were stored at −80°C. Bacterial functional diversity was assessed based on substrate-use profiles of 31 unique carbon substrates using Biolog EcoPlates™, an approach also known as community-level physiological profiling (CLPP) [34]; see supplementary methods for methodological details and Table S3 for the substrate list. Elemental composition analysis was used to test whether nutrient ratios differed among treatments (see supplementary methods). Carbon content was strongly correlated with maximum RFU yield (Pearson’s *r* = 0.91, *P* < .001) and was chosen as the main measure of culture biomass production (used as a proxy for productivity) for all analyses. DNA was extracted using phenol-chloroform DNA/RNA co-extraction [35] and further processed as described in the supplementary methods. In short, the V4–V5 region of the 16S rRNA gene was amplified using a 515f-Y and a 926r primer and sequenced on an Illumina Miseq-Short Read Sequencer (San Diego, CA, USA). Prokaryotic sequences were classified using a curated database constructed from SILVA132 [36] and PR2 [37] (to filter out eukaryotic 18S rRNA sequences) and assigned to amplicon sequence variants (ASV). Samples (2 ml) of inoculated stock cultures at 55 RFU after 36 h of inoculation before the start of the experiment as well as from all experimental units at the final sampling were fixed in 2% formalin and stored at 4°C to assess bacterial abundance. Prior to measurement, samples were filtered (50 μm mesh size), stained using SybrGreen I (10× final conc., Invitrogen, Thermo Fisher Scientific Inc.), and incubated in the dark for 30 min. Cells were enumerated on an Accuri C6 flow cytometer (BD Biosciences, Franklin Lakes, NJ, USA) after [38]. As we did not use additional measures for detachment of particle-attached bacteria, such as sonication or solvent treatment, enumeration yielded counts for free-living bacteria [39].

### Statistical analysis

Statistical analysis was done in R v4.2.2 [40]. To account for varying sequencing depth, ASV counts were rarefied to 3000 sequences per sample (100 iterations) [41]. The effective number of species (ENS_ASV_) and the effective number of substrates utilized (ENS_CLPP_) as proxies for alpha-diversity were calculated based on the Shannon index (*H′*) as ENS = *e^H’^* [42]. Bray–Curtis distances were obtained as a measure of beta-diversity and used to characterize communities using non-metric multidimensional scaling (NMDS). Shannon index and Bray–Curtis distances were calculated using the R package vegan [43] v2.6.4. Permutational multivariate analysis of variance (Adonis PERMANOVA, using the vegan package) was run using Bray–Curtis distances with 999 permutations. Pairwise comparisons were done using pairwiseAdonis v0.4.1 to evaluate differences in beta-diversity between species and geographical origins; *P* values were adjusted using the Bonferroni method. The net biodiversity effect (NBE) for each variable of interest was calculated separately for each polyculture sample by subtracting the mean of the value in the respective included monocultures from the value in the polyculture following [4]. For example, the NBE for biomass is as follows:


$${\mathrm{NBE}}_{\mathrm{biomass}}={\mathrm{biomass}}_{\mathrm{polyculture}}-\mathrm{mean}\left({\mathrm{biomass}}_{\mathrm{component}\ \mathrm{monocultures}}\right)$$

Differences in ENS_ASV_ and ENS_CLPP_, biomass, and free-living bacterial abundance between diatom diversity treatments were assessed by Kruskal–Wallis and Dunn post-hoc tests (Holm adjustment). Structural equation modeling (SEM) using the R package “piecewiseSEM” (v2.1.2, [44]) allowed for more integrated inferences regarding the relationships among multiple variables. The fit of alternative SEM models (with/without interaction terms, with/without each variable) was assessed based on AICc; the SEM data shown are based on the overall best fit model. To test if certain bacterial taxa had pronounced effects on biomass, Spearman correlations between relative abundances of each bacterial group (at different taxonomic levels, from genus to class) and culture biomass were run using adjusted *P* values for multiple comparisons (Benjamini–Hochberg) within taxonomic levels. Statistical significance for all analyses was defined at α < 0.05.

## Results

### Diatom species harbor taxonomically but not functionally distinct microbiomes

**Figure 1:**
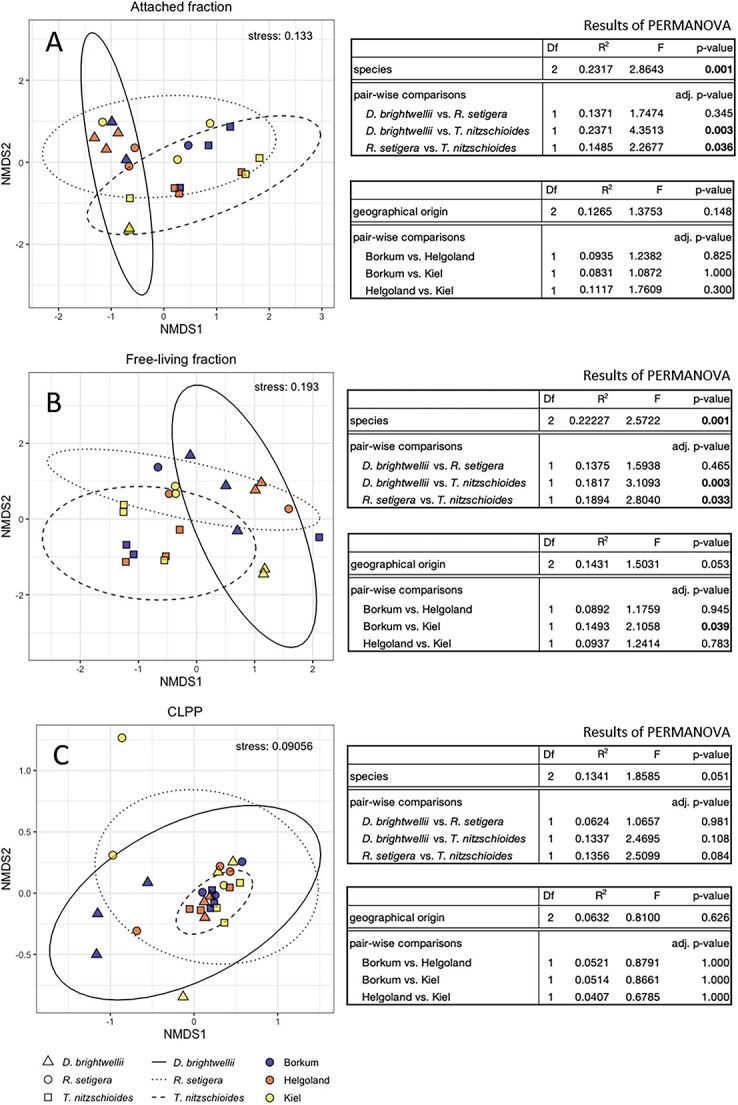
Bacterial community analysis by diatom strain. NMDS plots for diatom monocultures based on ASV of the attached (A) and free-living community (B) and physiological profiles/CLPP (C); ellipses show clustering by diatom species; tables contain respective results for PERMANOVA and pairwise comparisons across species/geographical origins.

The bacterial community in both the attached and free-living fractions was dominated by the classes *Alphaproteobacteria*, *Bacteroidia*, and *Gammaproteobacteria*, with dominance of the families *Rhodobacteraceae* and *Flavobacteracea* in the attached fraction and of *Rhodobacteraceae*, *Flavobacteracea*, and *Sphingomonadaceae* in the free-living fraction. Relative abundances of the most frequent taxa within the diatom microbiomes are shown in Figs S3–S6. The composition of associated bacteria did not show a marked difference between the original laboratory cultures and the inoculated cultures in the late exponential/early stationary phase (Fig. S7). NMDS analyses of diatom monocultures showed distinct compositional clusters of the associated microbiomes by diatom species in the attached and free-living bacterial fractions (Fig. 1A and B). Beta-diversity based on Bray–Curtis distances differed significantly between host species (PERMANOVA pseudo-*F* = 2.86; *P* = .001 for the attached; and PERMANOVA pseudo-F = 2.57; *P* = .001 for the free-living fraction, respectively, Fig. 1A and B). Differences in beta-diversity between geographical origins remained below the significance threshold, indicating species-specific differences in microbiome composition in both bacterial fractions regardless of culture origins. Certain geographical strains, however, did exhibit clear separation in microbiomes from their conspecifics from other origins (e.g. *D. brightwellii* isolated from Kiel in both fractions). Regarding CLPP, NMDS analysis and PERMANOVA on diatom monocultures revealed neither differences between diatom species nor geographical origin (Fig. 1C; see Discussion and Fig. S8 regarding substrate-use profiles).

**Figure 2:**
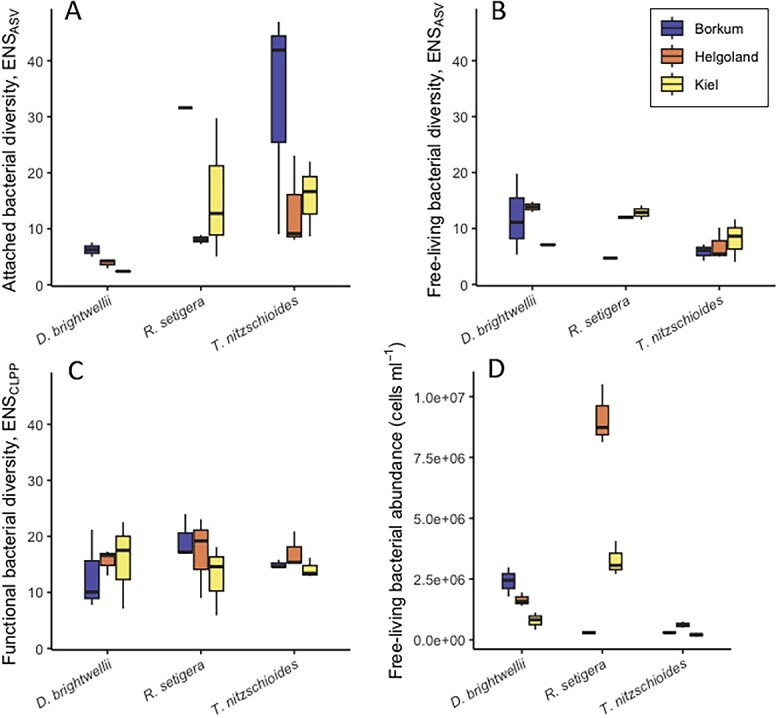
Bacterial diversity and abundance in diatom monocultures. Attached bacterial diversity (A), free-living bacterial diversity (B), functional bacterial diversity/CLPP (C), and free-living bacterial abundance (D).

Among host monocultures of *R. setigera* and *T. nitzschioides*, ASV diversity (ENS_ASV_) was higher in the attached fraction than in the free-living fraction (Fig. 2A and B). *D. brightwellii* monocultures exhibited the lowest ENS_ASV_ within the attached community (Fig. 2A) (Kruskal–Wallis χ^2^_2_ = 13.12; *P* = .001), providing some evidence for the presence of species-specific differences in the diversity of the attached community in addition to compositional aspects. ENS_ASV_ of the free-living community did not differ significantly between species (Kruskal–Wallis χ^2^_2_ = 5.18; *P* = .075), and there were no significant differences in either fraction between geographic origins (attached fraction, Fig. 2A: Kruskal–Wallis χ^2^_2_ = 2.92; *P* = .232; free-living fraction, Fig. 2B: Kruskal–Wallis χ^2^_2_ = 2.03; *P* = .363). CLPP diversity (ENS_CLPP_) did not differ significantly between monoculture species or strains (Fig. 2C, Kruskal–Wallis χ^2^_2_ = 1.15; *P* = .564 for differences between species, and Kruskal–Wallis χ^2^_2_ = 1.03; *P* = .598 for differences between geographical origins), indicating functional redundancy across phylogenetically divergent microbiomes.

### Diatom species and strain richness increase culture biomass production but not attached bacterial diversity

**Figure 3:**
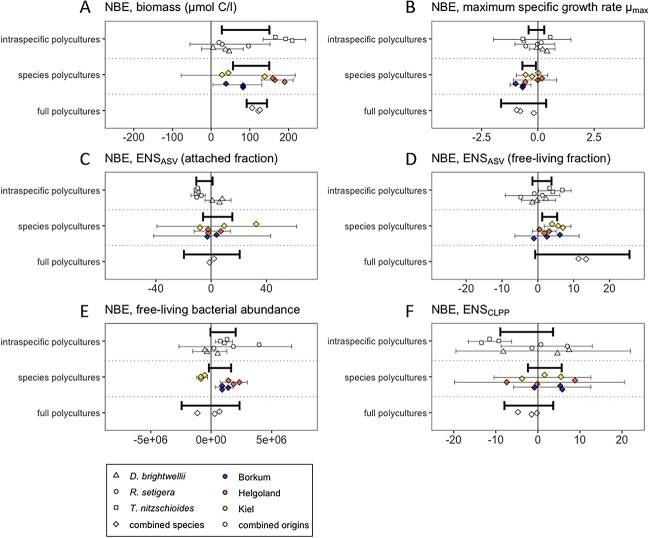
Effects of diatom biodiversity on biomass and bacterial diversity and abundance. Net biodiversity effects (NBE) of the three diatom polyculture treatments on biomass (A), maximum specific growth rate (B), alpha-diversity ENS_ASV_ of the attached (C) and free-living fractions (D), free-living bacterial abundance (E), and bacterial physiological diversity ENS_CLPP_ (F); thick black bars indicate the 95% confidence interval for the overall NBE in each diversity level; thin bars indicate 95% confidence intervals for NBE by individual species/strain treatment combinations; positive NBE values not overlapping zero indicate positive effects of increased diversity.

The effect of diatom diversity can be expressed in terms of the NBE, which explicitly shows the magnitude of diversity effects on each response variable in polycultures relative to their component monocultures. Biodiversity effects of the polyculture treatments are summarized as thick black bars (95% confidence intervals) in Fig. 3, such that bars not overlapping zero indicate significant diversity effects. The overall NBE on biomass was significantly positive and similar in magnitude in the three polyculture treatments (Fig. 3A), indicating a comparable positive effect of intraspecific and species diversity on biomass production. This was supported by an equivalent biomass increase in intraspecific and species polycultures compared with monocultures (Fig. S9A). There was no overall effect of intraspecific or species diversity on the diversity of the attached bacterial community (Fig. 3C and Fig. S9C). However, there was an overall positive effect of increased species diversity and the maximum diversity treatment (“full polycultures”) on the diversity of the free-living bacterial fraction (Fig. 3D and Fig. S9D). None of the polyculture treatments showed a significantly positive or negative overall NBE regarding bacterial abundance (Fig. 3E and Fig. S9E) or CLPP diversity (Fig. 3F and Fig. S9F), and the different diversity treatments did not differ in stoichiometric ratios (Fig. S10). Regarding the effects of distinct treatment combinations within the three diversity treatments (denoted by thin black bars in Fig. 3), there was evidence of a negative effect of intraspecific richness on bacterial taxonomic and functional diversity specific to certain species/strain combinations [e.g. negative NBE for ENS_CLPP_ and ENS_ASV_ (attached fraction) in intraspecific *T. nitzschioides* polycultures and for ENS_ASV_ (attached fraction) in intraspecific *R. setigera* polycultures, Fig. 3C and F]. Conversely, intraspecific *T. nitzschioides* polycultures showed a positive NBE for free-living bacterial diversity and abundance (Fig. 3D and E).

### Integrating phytoplankton and bacterial diversity effects on biomass using structural equation modeling

**Figure 4:**
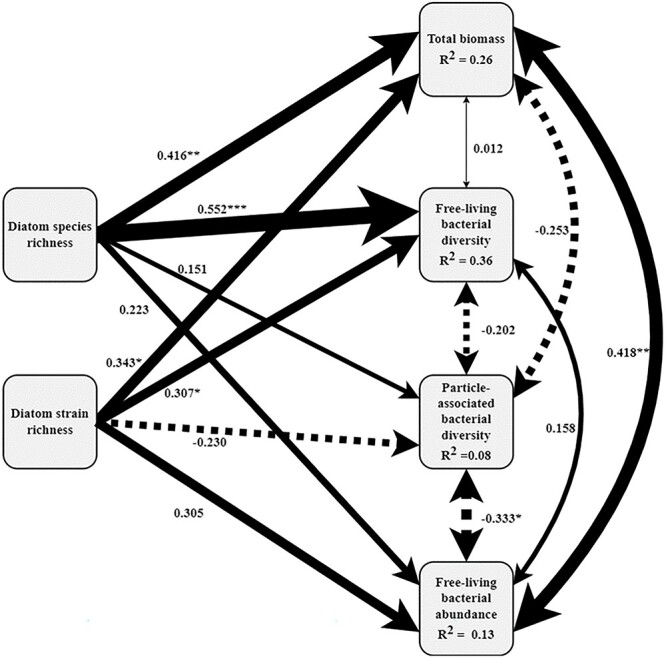
Structural equation modeling (SEM) pathways showing relationships among key variables of interest; arrow size is proportional to the magnitude of standardized coefficients, which are also shown as numbers adjacent to arrows; stars represent significant effects (^***^P < .001; ^**^P < .01; ^*^P < .05); single-headed arrows represent hypothesized unidirectional relationships (linear regressions), double-headed arrows represent hypothesized bidirectional/correlative relationships. Solid and dashed lines represent positive and negative effects, respectively.

SEM provides an integrated multivariate analysis of both direct and indirect effects of diatom diversity on biomass, as mediated by bacterial diversity (Fig. 4). The causal pathways we hypothesized are shown as single-headed arrows in Fig. 4. The analysis confirmed strong effects of diatom species and strain richness on biomass and free-living bacterial diversity, as well as a lack of an association between diatom diversity and attached bacterial diversity. SEM models including interactions between diatom species and strain richness had a poorer fit based on AICc scores, indicating that only additive and not interactive diversity effects were important. Similarly, removing CLPP enhanced model fit, confirming that substrate-use diversity was neither related to diatom diversity nor biomass production. The relative magnitudes of effects in the SEM pathways suggest that bacterial taxonomical and functional diversity did not clearly mediate diversity-functioning patterns. Thus, the indirect effects of diatom diversity on biomass were of minor importance in our experimental system.

Additionally, SEM can reveal correlations between the different response variables in this study, providing further insights into the relationship between diatom and bacterial communities (double-headed arrows in Fig. 4). Specifically, our results show that diatom biomass was significantly related to free-living bacterial abundance but not to attached or free-living bacterial diversity or substrate-use diversity. Additionally, a negative relationship between attached bacterial diversity and free-living bacterial abundance emerged from the analysis.

### Productivity is not significantly correlated with the abundance of distinct bacterial taxa across treatments

A Spearman correlation analysis across all treatments adjusted for multiple comparisons revealed no significant association of overall productivity with the relative abundance of specific bacterial taxa in the attached and free-living fractions (Table S4). An analysis conducted separately for each host species (combining monocultures and intraspecific polycultures) to assess potential species-specificity in enhancing productivity, correspondingly, did not show any distinct associations (Table S5A–F). Thus, differences in productivity across treatments and within mono- and intraspecific polycultures of a single species could not be attributed to the relative abundance of specific bacterial taxa present.

## Discussion

The experimental results provided mixed support for the initial hypotheses regarding diatom-bacteria relationships and ecosystem functioning. Bacterial community composition in most cases was not specific to distinct strains but tended to be species-specific, providing partial support for H1. Intraspecific and species richness had equal effects on increasing biomass production, which clearly supported H2. There was limited support for H3 since diatom diversity affected the diversity of the free-living but not of the attached bacterial communities. In disagreement with H4, the analyses yielded no relationship between bacterial diversity and community biomass production.

### Host-specificity of microbiomes

Differences in the phylogenetic composition of microbiomes between host species (Fig. 1) correspond with numerous findings in the literature regarding the phylogenetic signal of microbial symbionts [24, 45, 46]. However, the consistent presence of certain bacterial taxa across all strains is also consistent with the idea of a “core microbiome” for diatom species [47]. We did not observe host-specific differences in bacterial CLPP (Fig. 1C), which is consistent with reports of high functional redundancy in phylogenetically distinct bacterial communities [48, 49]. Most cultures had high usage of certain carbohydrates (e.g. cellobiose, cyclodextrin) and amino acids (e.g. arginine, asparagine) and low use of other compounds (e.g. xylose, pyruvic acid methyl ester) (Fig. S8); these similarities could have driven the overall similarity in multivariate CLPP profiles across treatments. CLPP composition of *D. brightwellii* diverged between Borkum and the other locations, possibly indicating minor differences between individual strains rather than consistent differences in physiological profiles across all strains of a species. Although the CLPP assay can provide a general insight into the metabolic range of a bacterial community, the array of substrates used for CLPP assessment may not comprehensively represent the composition of organic compounds released by diatoms, typically comprised of high-molecular-weight molecules requiring specific enzymatic capabilities for degradation [50–52]. The decomposition of host-specific compounds is often compartmentalized within bacterial communities, shaping bacterial community composition and spatial host-epibiont interactions [50, 53, 54]. Thus, although functional redundancy across taxa is a fitting explanation for our data, it is possible that the phylogenetically distinct bacterial communities in our diatom cultures possess distinct specialized metabolic functions not detected by our methods.

### Diversity linkages across domains

Bacterial diversity of the attached fraction differed between host monocultures (Fig. 2A), suggesting that host-specificity of the associated microbiomes involved aspects of bacterial diversity in addition to mere compositional differences. ASV and CLPP diversity were not correlated (Fig. S11), which provided further support for the presence of functional redundancy within the assessed microbiomes [48]. Diatom diversity had a positive NBE on the diversity of the free-living bacterial community, which increased from intraspecific to species and full polycultures (Fig. 3D), providing limited evidence for a positive link between diversity across domains. NBE values for the attached bacterial community did not show the same tendency; conversely, NBE values were negative regarding the diversity of the attached fraction in intraspecific polycultures of *T. nitzschioides* and *R. setigera* (Fig. 3C). Generally, NBE, especially regarding attached bacterial diversity and free-living bacterial abundance (Fig. 3C and E), varied substantially based on the species and strain combination, suggesting that diversity effects in this system were contingent on diatom community composition, in some cases more than on diversity levels per se. We estimated the relative abundances of each species in the 3-species and full polycultures, which revealed a fairly even distribution, i.e. that no species (and thus its specific microbiome) was completely outcompeted during the experiment (Fig. S12). However, it was not possible to determine the relative abundances of each strain in the intraspecific polycultures, so it is unclear whether some of the diatom strains associated with high monoculture ASV diversity were outcompeted by other diatom strains in polyculture, leading to a relative deficit in ASV diversity in intraspecific polycultures compared with the corresponding monocultures. Species polycultures, however, did not have negative NBE values (i.e. confidence intervals for NBE overlapped zero for all species polyculture combinations in Fig. 3C). This suggests a potentially divergent influence of intra- and interspecific host diversity on the composition of the attached communal microbiome. The reduced ASV diversity in certain intraspecific polycultures relative to monocultures indicates that microbiome diversity did not generally increase with host diversity or that this effect was possibly overruled by synergistic host-specific selection mechanisms in certain host combinations [19]. There are several reasons for lower diversity in intraspecific polycultures, e.g. (i) there could have been a selection effect where higher diatom strain richness meant a higher chance of introducing a dominant bacterium or (ii) diatoms exert a synergistic effect such that when multiple genotypes co-occur, they can more effectively structure their microbiomes, converging more toward a host-specific core microbiome [47]. Other mechanisms at play in nature could be allelopathy, predation, or competition among specific bacteria [17, 55, 56], which could impact the communal microbiome when particular intraspecific strains of diatoms are brought together. Host-specific microbiomes can also become less rich and more specific over time [19], so it is possible our ASV diversity results could differ along with experimental duration. Rarefaction of ASV data for diversity assessment could have induced a methodical bias, preventing the detection of rare ASVs [57] in polycultures. CLPP analysis confirmed the lack of a generalized increase in bacterial (phylogenetic or physiological) diversity with increasing host diversity (Figs 3F and S9F). However, the loss of rare taxa when transferring to CLPP plates is also possible.

### Diatom diversity and productivity

A positive relationship between diatom diversity and productivity (Figs 3A and S9A) matches findings reported in various ecosystems [1–3, 5, 27]. The positive diversity-productivity relationship is often found to decelerate at high diversity, which is attributed to a saturating effect regarding resource partitioning and functional redundancy in communities exceeding a certain diversity threshold, limiting the positive effects of a further increase in diversity [10, 58]. There were equivalent positive effects of intraspecific and species richness on biomass accumulation (Figs 3A and 4). This may be due to a high functional trait diversity among individuals within a species, which can be similar in magnitude to the trait diversity among distinct species [10, 59], causing similar degrees of complementarity effects in resource use [4] (or, alternatively, similar magnitudes of selection effects for species and strain richness). Intraspecific differences in diatom growth rates indicate high trait variability among intraspecific strains (Fig. S13). However, for the subset of treatments where partitioning NBE into its components was possible (complementarity effects versus selection effects [4]), we found that complementarity effects were positive and selection effects were variable but centered around zero (Fig. S14), consistent with similar findings in artificial benthic diatom communities [16, 27]. In other words, these polycultures were not generally dominated by strains with the highest biomass, but rather each constituent species tended to have higher biomass in polyculture than expected based on monoculture biomass. Comparable C:N and C:P ratios between the diversity treatments suggested, however, that the enhanced accumulation of biomass in polycultures did not arise due to increased efficiency in the use of the macronutrients nitrogen and phosphorus (Fig. S10); i.e. as greater C per unit N or P would imply greater resource use efficiency of biomass production [60, 61]. Other possible factors might be enhanced efficiency in the use of micronutrients or light, as well as resource-independent facilitation effects between interaction partners [16]. The magnitude of intraspecific diversity effects on productivity in this study clearly underlines the importance of incorporating intraspecific diversity in BEF research.

### Diversity and productivity across domains

The increase in productivity in intraspecific and species polycultures (positive NBE for biomass, Fig. 3A), in contrast to the decrease in attached bacterial diversity in intraspecific *T. nitzschioides* and *R. setigera* polycultures (negative NBE for ENS_ASV_, Fig. 3C), emphasizes the decoupling of bacterial diversity from the diatom diversity-productivity relationship. Matching reported outcomes of varying diatom diversity in benthic diatom-bacteria biofilms [27], the results of the SEM confirmed the lack of evidence for a positive diversity-productivity relationship across taxonomic domains for both the attached and the free-living bacterial communities (Fig. 4). Although bacterial diversity has been found to improve the productivity of terrestrial autotrophs in field experiments [7, 62], there is some evidence that this beneficial relationship is mostly mediated by a stabilizing effect on productivity under environmental fluctuation and disturbance [63, 64]. Environmental variability was absent in this controlled laboratory experiment; thus, the role of bacteria may differ with more field-realistic levels of environmental heterogeneity. Jackrel *et al.* [19] found positive fitness effects of less rich, more host-specific associated microbiomes on green algae. Certain bacterial taxa can enhance the growth of their phytoplankton host [20, 65], while others exert antagonistic effects [66, 67] or compete with algae for nutrients [68, 69]. The encounter of more divergent host-specific microbiomes or an increase in overall bacterial diversity might therefore increase the risk of antagonistic effects or competition, which might offset the potential benefits of a larger range of microbial functional traits. Correspondingly, the results of this study suggest that the identity and distinct functional profile of affiliated bacteria may be more relevant for host productivity than mere bacterial diversity.

### Bacterial taxonomy and productivity

Microbiomes were predominantly composed of known phycosphere taxa (e.g. *Alphaproteobacteria*, including *Rhodobacteraceae*, and various *Gammaproteobacteria* [25, 47]. Although correlation tests did not reveal significant relationships with bacterial taxa and community function after *P*-adjustment (Tables S4 and S5), the relative abundances of genera within the families *Rhizobiaceae* and *Rhodobacteraceae* in both fractions tended to show a positive association with biomass, while taxa within the order *Flavobacteriales* tended toward a negative association with biomass in the attached fraction and toward a positive association in the free-living fraction. Matching these findings, *Rhizobiaceae* have been found to enhance growth in microalgae [70], while different members of the *Rhodobacteraceae* family (“Roseobacter clade”) are typically involved in mutualistic interactions with phytoplankton [71, 72], often supplying Vitamin B12 to their algal host [73], with some evidence for a potential switch to antagonistic interactions in aging algal populations [74, 75]. *Flavobacteriales* are frequently found in association with diatom blooms and consume diatom lysate, suggesting an ability to invade and degrade diatom cells [76]. *Flavobacteriales* are designated as key marine polymer degraders, while *Rhodobacteraceae* are specialized to consume labile organic components of marine algal blooms [77, 78]. An increase in abundance of *Flavobacteriales* at high diatom biomass and substrate availability and a subsequent drop in diatom biomass under cell degradation depending on the stage of interaction (potentially also reflected in an attached vs. free-living lifestyle) can explain the existence of both positive and negative associations with culture biomass. Increased relative abundances of genera in the orders *Oceanospirillales* (important Vitamin B12 producers associated with diatom blooms [79, 80]) and *Alteromonadales* (specifically *Colwelliaceae* and *Marinobacteraceae*, both known degraders of phytoplankton-derived carbon compounds [81]), *Arcobacter* (a genus within *Campylobacterales*, for which a selective allelopathic effect on specific diatom species has been proposed [27]), and *Bdellovibrionales* (predatory bacteria [82]) in both fractions showed a tendency toward a negative correlation with culture biomass. Bacterial taxonomic annotation for correlations was only determined to genus level within this study, as this reduces the degree of multiple comparisons and because species-level annotation was not always possible. A lack of a statistically significant correlation robust to *P* value adjustment between productivity and the relative abundance of specific bacterial taxa (Tables S4 and S5) could thus be due to substantial differences in functional traits at a lower taxonomic level, i.e. between bacterial species or intraspecific bacterial strains [83]. Complex community interactions within diatom-associated microbiomes influencing productivity as well as the lack of environmental fluctuation in the study may additionally obscure direct productivity-enhancing effects of single taxa.

## Conclusions and future directions

In summary, these results contribute to our understanding of marine ecosystem functioning by showing that diatom species and strain diversity have strong positive effects on biomass; however, in contrast to our expectations, these biodiversity effects were overall not strongly moderated by host-associated bacterial diversity (either taxonomic diversity or substrate-use diversity). While some of our results validate previous work (e.g. species-specificity of diatom microbiomes), our results also open new questions. Specifically, we find stronger effects of diatom diversity on free-living bacterial diversity than particle-associated bacterial diversity; thus, further research is needed to resolve questions about the mechanisms underlying this potential disparity. The absence of any clear effect of bacterial diversity on biomass production also raises further questions for future research (e.g.: Is this due to a net cancelation of positive and negative bacteria-diatom interactions that comes with increasing diversity? How does increasing bacteria/diatom diversity influence the total flux of distinct bioactive metabolites/infochemicals, as well as the capacity to metabolize complex carbon substrates, that mediate diatom-bacteria interactions?). Furthermore, we suggest that incorporating environmental fluctuations and disturbances resembling natural conditions will be necessary to gain insights into the mechanisms and complex interactions at play impacting phytoplankton productivity and resilience under ongoing global change.

## Supplementary Material

4_Jacob_et_al-ISME_Comms_supplement-March_26,2024_ycae046

## Data Availability

The 16S metabarcoding data for this study have been deposited in the European Nucleotide Archive (ENA) at EMBL-EBI under accession number PRJEB60520 (https://www.ebi.ac.uk/ena/browser/view/PRJEB60520). All other relevant data and code is deposited on Zenodo (https://doi.org/10.5281/zenodo.10817693).
