# Supplementary material for: Cross-domain diversity effects: linking diatom species richness, intraspecific richness, and biomass production to host-associated bacterial diversity
Source: ISME Commun. 2024 Mar 29;4(1):ycae046. doi: 10.1093/ismeco/ycae046 (PMC11334582; doi:10.1093/ismeco/ycae046)
Supplement: 4_Jacob_et_al-ISME_Comms_supplement-March_26,2024_ycae046 [file 4_jacob_et_al-isme_comms_supplement-march_26,2024_ycae046.docx]

Supplementary material for:

**Cross-domain diversity effects: linking diatom species richness, intraspecific richness, and biomass production to host-associated bacterial diversity**

Marrit Jacob^1,2*^, Patrick K. Thomas^1,3*^, Helge-Ansgar Giebel^1^, Sara Billerbeck^1^, Meinhard Simon^1^, Maren Striebel^1^, Leon Dlugosch^1^

^1^ Institute for Chemistry and Biology of the Marine Environment (ICBM), School of Mathematics and Science, Carl von Ossietzky Universität Oldenburg, Ammerländer Heerstraße 114-118, 26129 Oldenburg, Germany

^2^ Marine Chemistry, Department of Biology and Chemistry, University of Bremen, James-Watt-Straße 1 (BIOM), 28359 Bremen, Germany

^3^Department of Aquatic Ecology, Swiss Federal Institute of Aquatic Science and Technology (EAWAG), 133 Überlandstrasse, 8600 Dübendorf, Switzerland

**Contents:**

Supplementary tables: Table S1-S5

Supplementary figures: Fig. S1-S14

Supplementary methods

# Supplementary tables

**Table S1**. Nucleotide substitutions per site derived based on neighbor-joining tree using a Tamura-Nei distance model from 18S analysis of the diatom strains.


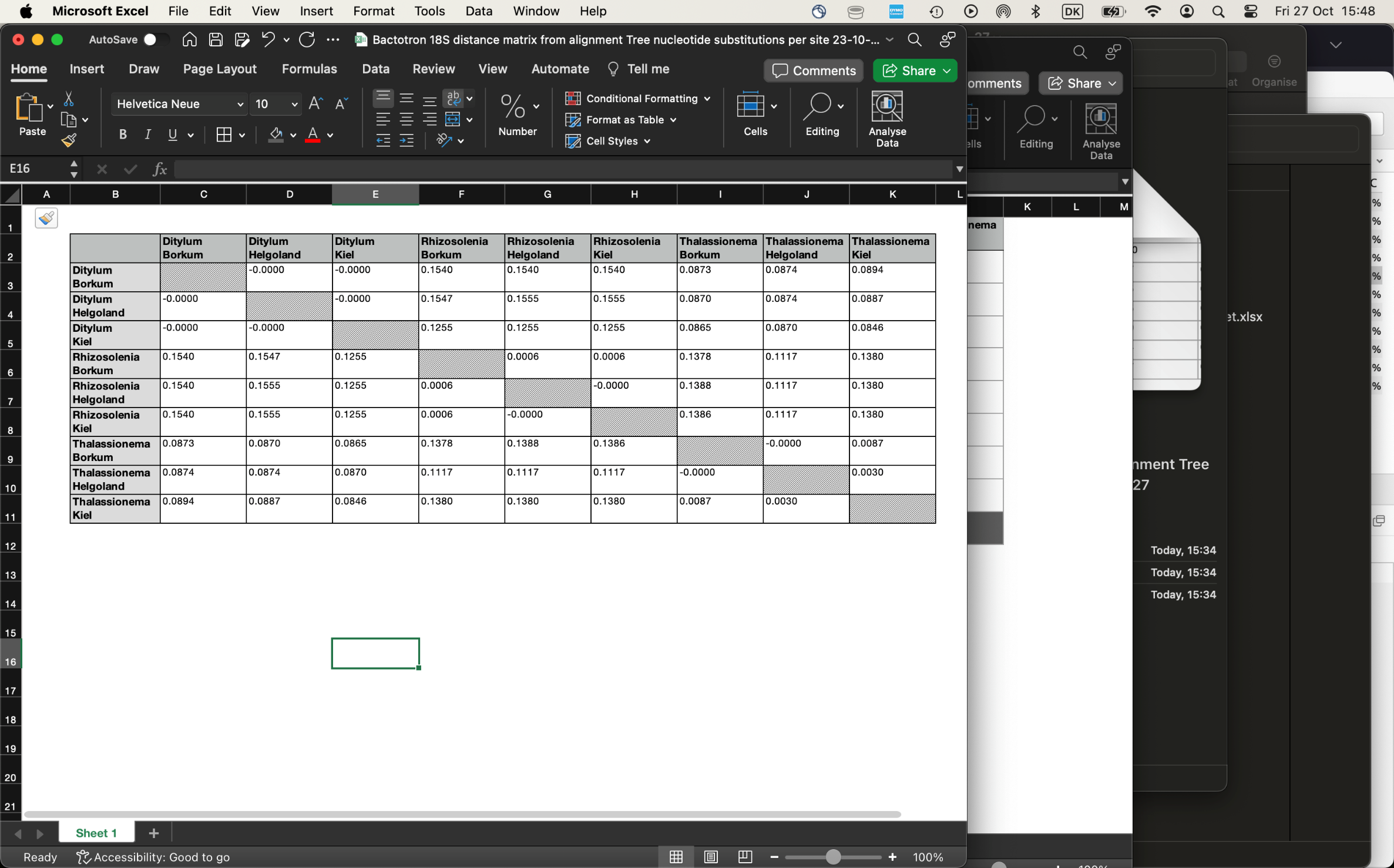


**Table S2.** Treatment combinations for experimental manipulation of species and strain richness. All combinations were run as triplicates and biomass/growth/CLPP data is available for all 3 replicates. However, some 16S samples were lost during processing, reducing sample size for treatments: DK (n = 2), RB (n = 1), RH (n = 2), full combination (n = 2).

| Diversity treatment | # of individual treatment combinations | Individual treatment combinations | Overall strain richness | Intraspecific  richness | Species  richness |
| --- | --- | --- | --- | --- | --- |
| A: monocultures | 9 | **1. DB**: *D. brightwellii* - Borkum  **2. DH**: *D. brightwellii* - Helgoland  **3. DK**: *D. brightwellii* - Kiel  **4. RB**: *R. setigera* - Borkum  **5. RH**: *R. setigera* - Helgoland  **6. RK**: *R. setigera* - Kiel  **7. TB**: *T. nitzschioides* - Borkum  **8. TH**: *T. nitzschioides* - Helgoland  **9. TK**: *T. nitzschioides* - Kiel | 1 | 1 | 1 |
| B: intraspecific polycultures | 3 | **1. DBDHDK**: *D. brightwellii* - Borkum  *D. brightwellii* - Helgoland  *D. brightwellii* - Kiel  **2. RBRHRK**: *R. setigera* - Borkum  *R. setigera* - Helgoland  *R. setigera* - Kiel  **3. TBTHTK**: *T. nitzschioides* - Borkum  *T. nitzschioides* - Helgoland  *T. nitzschioides* - Kiel | 3 | 3 | 1 |
| C: species polycultures | 3 | **1. DBRBTB**: *D. brightwellii* – Borkum  *R. setigera* - Borkum  *T. nitzschioides* - Borkum  **2. DHRHTH**: *D. brightwellii* - Helgoland  *R. setigera* - Helgoland  *T. nitzschioides* - Helgoland  **3. DKRKTK**: *D. brightwellii* - Kiel  *R. setigera* - Kiel  *T. nitzschioides* - Kiel | 3 | 1 | 3 |
| D: polyculture of  all strains | 1 | **full combination of all 9 strains:**  *D. brightwellii* - Borkum  *D. brightwellii* - Helgoland  *D. brightwellii* - Kiel  *R. setigera* - Borkum  *R. setigera* - Helgoland  *R. setigera* - Kiel  *T. nitzschioides* - Borkum  *T. nitzschioides* - Helgoland  *T. nitzschioides* - Kiel | 9 | 3 | 3 |

**Table S3.** Substrates provided on Biolog EcoPlates™, adapted from [1]


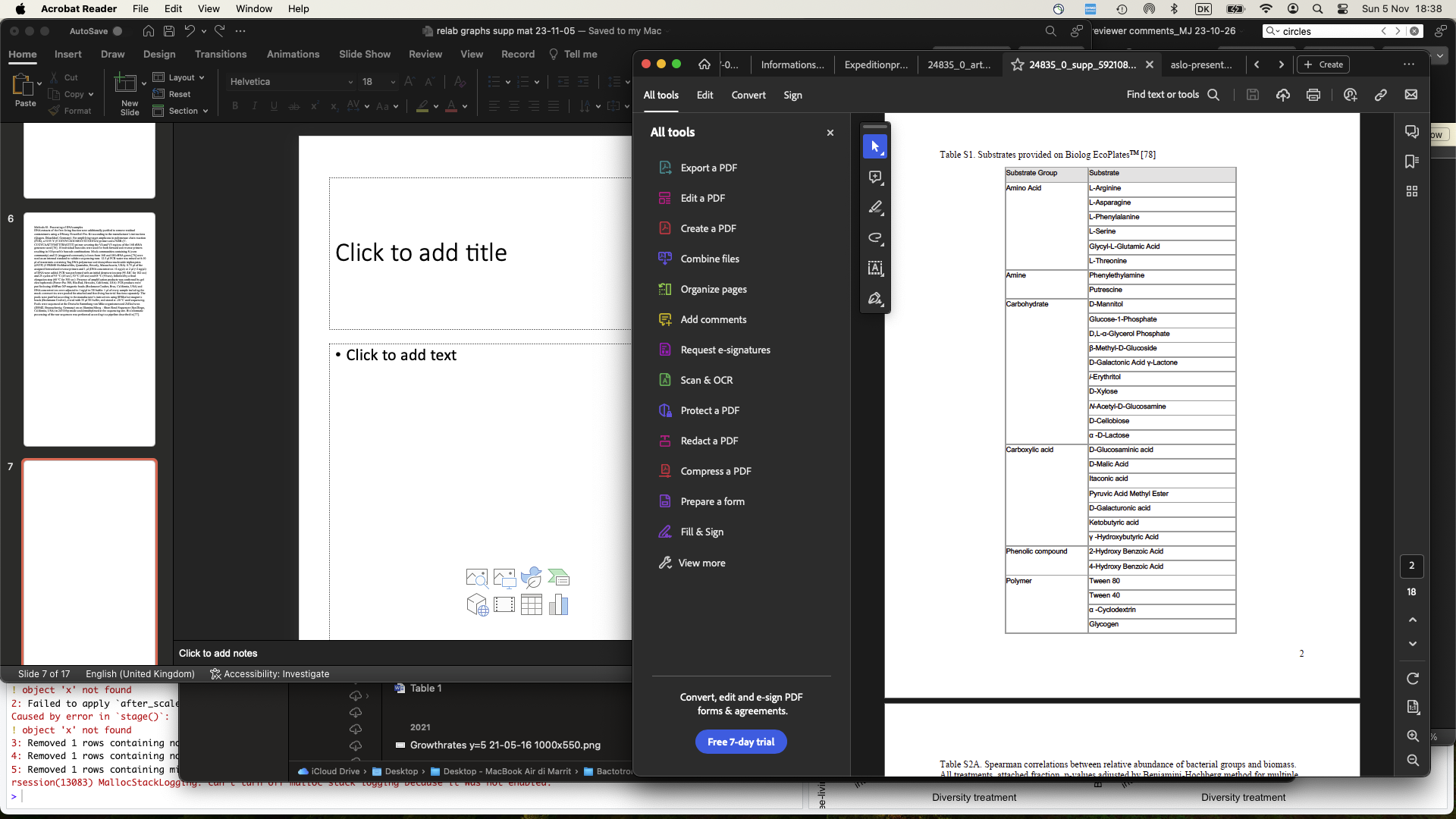


**Table S4A.** Spearman correlations between relative abundance of bacterial groups and biomass. All treatments, attached fraction. p-values adjusted by Benjamini-Hochberg method for multiple comparisons.

| Taxonomic level |  | correlation coefficient | p-value | adjusted  p-value |
| --- | --- | --- | --- | --- |
| Genus | Bacteroidia;Cytophagales;Cyclobacteriaceae;Reichenbachiella | -0,33 | 0,035 | 0,433 |
|  | Deltaproteobacteria;Deltaproteobacteria SAR324 clade(Marine group B);Deltaproteobacteria SAR324 clade(Marine group B);Deltaproteobacteria SAR324 clade(Marine group B) | -0,42 | 0,006 | 0,364 |
|  | Bacteroidia;Cytophagales;Cyclobacteriaceae;Ekhidna | -0,33 | 0,036 | 0,433 |
|  | Alphaproteobacteria;Rhodobacterales;Rhodobacteraceae;Planktotalea | 0,36 | 0,022 | 0,433 |
|  | Deltaproteobacteria;Bdellovibrionales;Bdellovibrionaceae;OM27 clade | -0,33 | 0,035 | 0,433 |
|  | Bacteroidia;Flavobacteriales;Flavobacteriaceae;[Polaribacter] huanghezhanensis | -0,37 | 0,017 | 0,433 |
|  | Gammaproteobacteria;Alteromonadales;Marinobacteraceae;Marinobacter | -0,33 | 0,038 | 0,433 |
|  | Campylobacteria;Campylobacterales;Arcobacteraceae;Arcobacter | -0,33 | 0,033 | 0,433 |
|  | Alphaproteobacteria;Rickettsiales;Rickettsiaceae;Candidatus Cryptoprodotis | -0,33 | 0,032 | 0,433 |
|  | Gammaproteobacteria;Alteromonadales;Colwelliaceae;Colwellia | -0,47 | 0,002 | 0,351 |
|  | Gammaproteobacteria;Oceanospirillales;Saccharospirillaceae;Oleispira | -0,34 | 0,030 | 0,433 |
|  | unknown Bacteria;unknown Bacteria;unknown Bacteria;unknown Bacteria | -0,34 | 0,028 | 0,433 |
|  | Deltaproteobacteria;PB19;unknown PB19;unknown PB19 | -0,42 | 0,006 | 0,364 |
|  | Alphaproteobacteria;Puniceispirillales;SAR116 clade;SAR116 clade | -0,34 | 0,029 | 0,433 |
|  | Gammaproteobacteria;Oceanospirillales;Halomonadaceae;Oceanospirillum | -0,34 | 0,029 | 0,433 |
|  | Clostridia;Clostridiales;Christensenellaceae;unknown Christensenellaceae | -0,31 | 0,047 | 0,499 |
|  |  |  |  |  |
| Family | Alphaproteobacteria;Rhodobacterales;Rhodobacteraceae | 0,32 | 0,045 | 0,299 |
|  | Bacteroidia;Cytophagales;Cyclobacteriaceae | -0,33 | 0,036 | 0,299 |
|  | Deltaproteobacteria;Deltaproteobacteria SAR324 clade(Marine group B);Deltaproteobacteria SAR324 clade(Marine group B) | -0,42 | 0,006 | 0,200 |
|  | Bacteroidia;Flavobacteriales;Crocinitomicaceae | -0,32 | 0,040 | 0,299 |
|  | Deltaproteobacteria;Bdellovibrionales;Bdellovibrionaceae | -0,33 | 0,035 | 0,299 |
|  | Gammaproteobacteria;Alteromonadales;Marinobacteraceae | -0,33 | 0,038 | 0,299 |
|  | Campylobacteria;Campylobacterales;Arcobacteraceae | -0,33 | 0,033 | 0,299 |
|  | Alphaproteobacteria;Rickettsiales;Rickettsiaceae | -0,40 | 0,010 | 0,245 |
|  | Gammaproteobacteria;Alteromonadales;Colwelliaceae | -0,47 | 0,002 | 0,193 |
|  | unknown Bacteria;unknown Bacteria;unknown Bacteria | -0,34 | 0,028 | 0,299 |
|  | Deltaproteobacteria;PB19;unknown PB19 | -0,42 | 0,006 | 0,200 |
|  | Alphaproteobacteria;Puniceispirillales;SAR116 clade | -0,34 | 0,029 | 0,299 |
|  | Bacteroidia;Chitinophagales;Saprospiraceae | -0,32 | 0,044 | 0,299 |
|  | Gammaproteobacteria;Oceanospirillales;Halomonadaceae | -0,34 | 0,029 | 0,299 |
|  | Clostridia;Clostridiales;Christensenellaceae | -0,31 | 0,047 | 0,299 |
|  |  |  |  |  |
| Order | Alphaproteobacteria;Rhodobacterales | 0,32 | 0,045 | 0,325 |
|  | Deltaproteobacteria;Deltaproteobacteria SAR324 clade(Marine group B) | -0,42 | 0,006 | 0,160 |
|  | Gammaproteobacteria;Alteromonadales | -0,41 | 0,009 | 0,160 |
|  | Campylobacteria;Campylobacterales | -0,33 | 0,033 | 0,291 |
|  | Alphaproteobacteria;Rickettsiales | -0,40 | 0,010 | 0,160 |
|  | unknown Bacteria;unknown Bacteria | -0,34 | 0,028 | 0,291 |
|  | Deltaproteobacteria;PB19 | -0,42 | 0,006 | 0,160 |
|  | Alphaproteobacteria;Puniceispirillales | -0,34 | 0,029 | 0,291 |
|  | Clostridia;Clostridiales | -0,31 | 0,047 | 0,325 |

**Table S4B.** Spearman correlations between relative abundance of bacterial groups and biomass. All treatments, free-living fraction. p-values adjusted by Benjamini-Hochberg method for multiple comparisons.

| Taxonomic level |  | correlation coefficient | p-value | adjusted  p-value |
| --- | --- | --- | --- | --- |
| Genus | Alphaproteobacteria;Rhodobacterales;Rhodobacteraceae;Planktotalea | 0,37 | 0,018 | 0,514 |
|  | Campylobacteria;Campylobacterales;Arcobacteraceae;Arcobacter | -0,40 | 0,009 | 0,375 |
|  | Gammaproteobacteria;Alteromonadales;Colwelliaceae;Colwellia | -0,51 | 0,001 | 0,120 |
|  | Alphaproteobacteria;Rhizobiales;Rhizobiaceae;Ahrensia | 0,43 | 0,005 | 0,269 |
|  | Gammaproteobacteria;Oceanospirillales;Halomonadaceae;Oceanospirillum | -0,48 | 0,002 | 0,139 |
|  | Gammaproteobacteria;Oceanospirillales;Nitrincolaceae;Amphritea | -0,39 | 0,011 | 0,375 |
|  |  |  |  |  |
| Famliy | Campylobacteria;Campylobacterales;Arcobacteraceae | -0,40 | 0,009 | 0,274 |
|  | Gammaproteobacteria;Alteromonadales;Colwelliaceae | -0,51 | 0,001 | 0,062 |
|  | Gammaproteobacteria;Oceanospirillales;Halomonadaceae | -0,48 | 0,002 | 0,072 |
|  |  |  |  |  |
| Order | Campylobacteria;Campylobacterales | -0,40 | 0,009 | 0,473 |
|  | Alphaproteobacteria;Rhizobiales | 0,36 | 0,021 | 0,522 |

**Table S5A.** *D. brightwellii* monocultures and intraspecific polycultures. Spearman correlations between relative abundance of bacterial groups and biomass. Attached fraction. p-values adjusted by Benjamini-Hochberg method for multiple comparisons.

| Taxonomic level |  | correlation coefficient | p-value | adjusted  p-value |
| --- | --- | --- | --- | --- |
| Genus | Alphaproteobacteria;Rhodobacterales;Rhodobacteraceae;Planktotalea | 0,57 | 0,014 | 0,441 |
|  | Gammaproteobacteria;Betaproteobacteriales;Burkholderiaceae;Paucibacter | 0,62 | 0,006 | 0,441 |
|  | Planctomycetacia;Planctomycetales;Gimesiaceae;unknown Gimesiaceae | -0,58 | 0,012 | 0,441 |
|  | Rhodothermia;Balneolales;Balneolaceae;Balneola | -0,47 | 0,050 | 0,630 |
|  | Gammaproteobacteria;Alteromonadales;Colwelliaceae;Colwellia | -0,56 | 0,017 | 0,441 |
|  | Deltaproteobacteria;Deltaproteobacteria SAR324 clade(Marine group B);Deltaproteobacteria SAR324 clade(Marine group B);Deltaproteobacteria SAR324 clade(Marine group B) | -0,56 | 0,015 | 0,441 |
|  | Alphaproteobacteria;Puniceispirillales;SAR116 clade;SAR116 clade | -0,47 | 0,050 | 0,630 |
|  | Gammaproteobacteria;Betaproteobacteriales;Burkholderiaceae;Cupriavidus | 0,54 | 0,019 | 0,441 |
|  | Gammaproteobacteria;Betaproteobacteriales;Burkholderiaceae;Variovorax | 0,50 | 0,036 | 0,630 |
|  |  |  |  |  |
| Family | Alphaproteobacteria;Rhodobacterales;Rhodobacteraceae | 0,55 | 0,020 | 0,280 |
|  | Gammaproteobacteria;Betaproteobacteriales;Burkholderiaceae | 0,59 | 0,012 | 0,280 |
|  | Planctomycetacia;Planctomycetales;Gimesiaceae | -0,58 | 0,012 | 0,280 |
|  | Rhodothermia;Balneolales;Balneolaceae | -0,47 | 0,050 | 0,413 |
|  | Gammaproteobacteria;Alteromonadales;Colwelliaceae | -0,56 | 0,017 | 0,280 |
|  | Deltaproteobacteria;Deltaproteobacteria SAR324 clade(Marine group B);Deltaproteobacteria SAR324 clade(Marine group B) | -0,56 | 0,015 | 0,280 |
|  | Alphaproteobacteria;Puniceispirillales;SAR116 clade | -0,47 | 0,050 | 0,413 |
|  |  |  |  |  |
| Order | Alphaproteobacteria;Rhodobacterales | 0,55 | 0,020 | 0,184 |
|  | Gammaproteobacteria;Betaproteobacteriales | 0,56 | 0,017 | 0,184 |
|  | Planctomycetacia;Planctomycetales | -0,58 | 0,012 | 0,184 |
|  | Rhodothermia;Balneolales | -0,47 | 0,050 | 0,293 |
|  | Alphaproteobacteria;Parvibaculales | 0,54 | 0,021 | 0,184 |
|  | Deltaproteobacteria;Deltaproteobacteria SAR324 clade(Marine group B) | -0,56 | 0,015 | 0,184 |
|  | Alphaproteobacteria;Puniceispirillales | -0,47 | 0,050 | 0,293 |

**Table S5B.** *D. brightwellii* monocultures and intraspecific polycultures. Spearman correlations between relative abundance of bacterial groups and biomass. Free-living fraction. p-values adjusted by Benjamini-Hochberg method for multiple comparisons.

| Taxonomic level |  | correlation coefficient | p-value | adjusted  p-value |
| --- | --- | --- | --- | --- |
| Genus | Bacteroidia;Flavobacteriales;Cryomorphaceae;unknown Cryomorphaceae | 0,47 | 0,043 | 0,324 |
|  | Bacteroidia;Flavobacteriales;Cryomorphaceae;Luteibaculum | 0,48 | 0,036 | 0,324 |
|  | Alphaproteobacteria;Rhodobacterales;Rhodobacteraceae;Planktotalea | 0,47 | 0,043 | 0,324 |
|  | Bacteroidia;Flavobacteriales;Flavobacteriaceae;Winogradskyella | 0,46 | 0,045 | 0,324 |
|  | Alphaproteobacteria;Rhodobacterales;Rhodobacteraceae;Marivita | 0,59 | 0,008 | 0,302 |
|  | Gammaproteobacteria;Oceanospirillales;Saccharospirillaceae;Reinekea | 0,49 | 0,033 | 0,324 |
|  | Gammaproteobacteria;Alteromonadales;Colwelliaceae;Colwellia | -0,51 | 0,025 | 0,324 |
|  | Gammaproteobacteria;Pseudomonadales;Pseudomonadaceae;Pseudomonas | 0,50 | 0,030 | 0,324 |
|  | Gammaproteobacteria;Betaproteobacteriales;Methylophilaceae;Methylotenera | 0,48 | 0,037 | 0,324 |
|  | Alphaproteobacteria;Sneathiellales;Sneathiellaceae;Sneathiella | -0,54 | 0,018 | 0,324 |
|  | Alphaproteobacteria;Sphingomonadales;Sphingomonadaceae;Sphingorhabdus | 0,53 | 0,018 | 0,324 |
|  | Alphaproteobacteria;Parvibaculales;PS1 clade;PS1 clade | 0,51 | 0,027 | 0,324 |
|  | Alphaproteobacteria;Caulobacterales;Caulobacteraceae;Brevundimonas | -0,50 | 0,028 | 0,324 |
|  | Alphaproteobacteria;Rhizobiales;Rhizobiaceae;Pseudahrensia | -0,65 | 0,003 | 0,302 |
|  | Deltaproteobacteria;Bdellovibrionales;Bacteriovoracaceae;unknown Bacteriovoracaceae | -0,46 | 0,045 | 0,324 |
|  | Alphaproteobacteria;Rhizobiales;Rhizobiaceae;Ahrensia | 0,51 | 0,025 | 0,324 |
|  | Alphaproteobacteria;Rhizobiales;Rhizobiaceae;unknown Rhizobiaceae | 0,50 | 0,030 | 0,324 |
|  | Gammaproteobacteria;Oceanospirillales;Halomonadaceae;Oceanospirillum | -0,58 | 0,009 | 0,302 |
|  | Alphaproteobacteria;Rhodobacterales;Rhodobacteraceae;Roseovarius | -0,58 | 0,009 | 0,302 |
|  |  |  |  |  |
| Family | Bacteroidia;Flavobacteriales;Cryomorphaceae | 0,58 | 0,009 | 0,305 |
|  | Gammaproteobacteria;Arenicellales;Arenicellaceae | 0,48 | 0,037 | 0,329 |
|  | Gammaproteobacteria;Alteromonadales;Colwelliaceae | -0,51 | 0,025 | 0,305 |
|  | Gammaproteobacteria;Pseudomonadales;Pseudomonadaceae | 0,50 | 0,030 | 0,305 |
|  | Alphaproteobacteria;Sneathiellales;Sneathiellaceae | -0,54 | 0,018 | 0,305 |
|  | Alphaproteobacteria;Parvibaculales;PS1 clade | 0,51 | 0,027 | 0,305 |
|  | Alphaproteobacteria;Caulobacterales;Caulobacteraceae | -0,50 | 0,028 | 0,305 |
|  | Gammaproteobacteria;Oceanospirillales;Halomonadaceae | -0,58 | 0,009 | 0,305 |
|  |  |  |  |  |
| Order | Gammaproteobacteria;Arenicellales | 0,48 | 0,037 | 0,320 |
|  | Gammaproteobacteria;Pseudomonadales | 0,48 | 0,038 | 0,320 |
|  | Bacilli;Bacillales | -0,47 | 0,044 | 0,320 |
|  | Alphaproteobacteria;Rhizobiales | 0,47 | 0,042 | 0,320 |
|  | Alphaproteobacteria;Sneathiellales | -0,54 | 0,018 | 0,320 |
|  | Alphaproteobacteria;Rhodospirillales | -0,51 | 0,024 | 0,320 |

**Table S5C.** *R. setigera* monocultures and intraspecific polycultures. Spearman correlations between relative abundance of bacterial groups and biomass. Attached fraction. p-values adjusted by Benjamini-Hochberg method for multiple comparisons.

| Taxonomic level |  | correlation coefficient | p-value | adjusted  p-value |
| --- | --- | --- | --- | --- |
| Genus | Bacteroidia;Flavobacteriales;Cryomorphaceae;unknown Cryomorphaceae | -0,50 | 0,043 | 0,699 |
|  | Bacteroidia;Flavobacteriales;Flavobacteriaceae;Winogradskyella | -0,51 | 0,035 | 0,699 |
|  | Bacteroidia;Flavobacteriales;Flavobacteriaceae;Croceibacter | -0,65 | 0,004 | 0,681 |
|  |  |  |  |  |
| Family | Bacteroidia;Flavobacteriales;Cryomorphaceae | -0,55 | 0,025 | 0,654 |
|  |  |  |  |  |
| Order | - |  |  |  |

**Table S5D.** *R. setigera* monocultures and intraspecific polycultures. Spearman correlations between relative abundance of bacterial groups and biomass. Free-living fraction. p-values adjusted by Benjamini-Hochberg method for multiple comparisons.

| Taxonomic level |  | correlation coefficient | p-value | adjusted  p-value |
| --- | --- | --- | --- | --- |
| Genus | Bacteroidia;Flavobacteriales;Crocinitomicaceae;Crocinitomix | -0,52 | 0,031 | 0,598 |
|  | Gammaproteobacteria;Oceanospirillales;Saccharospirillaceae;unknown Saccharospirillaceae | -0,59 | 0,012 | 0,539 |
|  | Deltaproteobacteria;PB19;unknown PB19;unknown PB19 | -0,53 | 0,030 | 0,598 |
|  | Bacteroidia;Flavobacteriales;Flavobacteriaceae;Maribacter | 0,52 | 0,031 | 0,598 |
|  | Deltaproteobacteria;Bdellovibrionales;Bacteriovoracaceae;unknown Bacteriovoracaceae | -0,53 | 0,030 | 0,598 |
|  | Gammaproteobacteria;Oceanospirillales;Saccharospirillaceae;Oleibacter | -0,60 | 0,010 | 0,539 |
|  | Gammaproteobacteria;Oceanospirillales;Nitrincolaceae;Amphritea | -0,66 | 0,004 | 0,537 |
|  |  |  |  |  |
| Family | Deltaproteobacteria;PB19;unknown PB19 | -0,53 | 0,030 | 0,874 |
|  |  |  |  |  |
| Order | Deltaproteobacteria;PB19 | -0,53 | 0,030 | 0,796 |

**Table S5E.** *T. nitzschioides* monocultures and intraspecific polycultures. Spearman correlations between relative abundance of bacterial groups and biomass. Attached fraction. p-values adjusted by Benjamini-Hochberg method for multiple comparisons.

| Taxonomic level |  | correlation coefficient | p-value | adjusted  p-value |
| --- | --- | --- | --- | --- |
| Genus | Bacteroidia;Cytophagales;Cyclobacteriaceae;Reichenbachiella | -0,55 | 0,013 | 0,374 |
|  | Verrucomicrobiae;Opitutales;Puniceicoccaceae;Puniceicoccaceae MB11C04 marine group | -0,47 | 0,037 | 0,387 |
|  | Bacteroidia;Cytophagales;Cyclobacteriaceae;Ekhidna | -0,66 | 0,002 | 0,174 |
|  | Bacteroidia;Cytophagales;Cyclobacteriaceae;unknown Cyclobacteriaceae | -0,52 | 0,017 | 0,374 |
|  | Alphaproteobacteria;Rhodobacterales;Rhodobacteraceae;Planktotalea | 0,49 | 0,028 | 0,374 |
|  | Bacteroidia;Flavobacteriales;Flavobacteriaceae;[Polaribacter] huanghezhanensis | -0,62 | 0,004 | 0,202 |
|  | Actinobacteria;Micrococcales;Microbacteriaceae;Candidatus Aquiluna | -0,46 | 0,043 | 0,409 |
|  | Campylobacteria;Campylobacterales;Arcobacteraceae;Arcobacter | -0,46 | 0,044 | 0,409 |
|  | Gammaproteobacteria;Oceanospirillales;Saccharospirillaceae;Reinekea | -0,48 | 0,031 | 0,374 |
|  | Gammaproteobacteria;Betaproteobacteriales;Burkholderiaceae;Burkholderiaceae RS62  marine group | -0,51 | 0,021 | 0,374 |
|  | Gammaproteobacteria;Alteromonadales;Colwelliaceae;Colwellia | -0,60 | 0,006 | 0,222 |
|  | Deltaproteobacteria;unknown Deltaproteobacteria;unknown Deltaproteobacteria;unknown Deltaproteobacteria | -0,49 | 0,029 | 0,374 |
|  | unknown Bacteria;unknown Bacteria;unknown Bacteria;unknown Bacteria | -0,66 | 0,001 | 0,174 |
|  | Deltaproteobacteria;PB19;unknown PB19;unknown PB19 | -0,49 | 0,027 | 0,374 |
|  | Deltaproteobacteria;Deltaproteobacteria SAR324 clade(Marine group B);Deltaproteobacteria SAR324 clade(Marine group B);Deltaproteobacteria SAR324 clade(Marine group B) | -0,49 | 0,027 | 0,374 |
|  | Gammaproteobacteria;Oceanospirillales;Saccharospirillaceae;Salinispirillum | -0,48 | 0,030 | 0,374 |
|  | Clostridia;Clostridiales;Christensenellaceae;unknown Christensenellaceae | -0,48 | 0,033 | 0,374 |
|  |  |  |  |  |
| Family | Bacteroidia;Flavobacteriales;Cryomorphaceae | -0,51 | 0,024 | 0,361 |
|  | Bacteroidia;Cytophagales;Cyclobacteriaceae | -0,60 | 0,006 | 0,176 |
|  | Verrucomicrobiae;Opitutales;Puniceicoccaceae | -0,47 | 0,037 | 0,361 |
|  | Campylobacteria;Campylobacterales;Arcobacteraceae | -0,46 | 0,044 | 0,389 |
|  | Gammaproteobacteria;Alteromonadales;Colwelliaceae | -0,60 | 0,006 | 0,176 |
|  | Deltaproteobacteria;unknown Deltaproteobacteria;unknown Deltaproteobacteria | -0,49 | 0,029 | 0,361 |
|  | unknown Bacteria;unknown Bacteria;unknown Bacteria | -0,66 | 0,001 | 0,129 |
|  | Deltaproteobacteria;PB19;unknown PB19 | -0,49 | 0,027 | 0,361 |
|  | Deltaproteobacteria;Deltaproteobacteria SAR324 clade(Marine group B);Deltaproteobacteria SAR324 clade(Marine group B) | -0,49 | 0,027 | 0,361 |
|  | Clostridia;Clostridiales;Christensenellaceae | -0,48 | 0,033 | 0,361 |
|  |  |  |  |  |
| Order | Bacteroidia;Cytophagales | -0,45 | 0,047 | 0,295 |
|  | Verrucomicrobiae;Opitutales | -0,47 | 0,037 | 0,295 |
|  | Gammaproteobacteria;Alteromonadales | -0,51 | 0,024 | 0,295 |
|  | Campylobacteria;Campylobacterales | -0,46 | 0,044 | 0,295 |
|  | Deltaproteobacteria;unknown Deltaproteobacteria | -0,49 | 0,029 | 0,295 |
|  | unknown Bacteria;unknown Bacteria | -0,66 | 0,001 | 0,083 |
|  | Deltaproteobacteria;PB19 | -0,49 | 0,027 | 0,295 |
|  | Deltaproteobacteria;Deltaproteobacteria SAR324 clade(Marine group B) | -0,49 | 0,027 | 0,295 |
|  | Clostridia;Clostridiales | -0,48 | 0,033 | 0,295 |

**Table S5F.** *T. nitzschioides* monocultures and intraspecific polycultures. Spearman correlations between relative abundance of bacterial groups and biomass. Free-living fraction. p-values adjusted by Benjamini-Hochberg method for multiple comparisons.

| Taxonomic level |  | correlation coefficient | p-value | adjusted  p-value |
| --- | --- | --- | --- | --- |
| Genus | Alphaproteobacteria;Rhodobacterales;Rhodobacteraceae;Planktotalea | 0,53 | 0,014 | 0,415 |
|  | Bacteroidia;Sphingobacteriales;Sphingobacteriales NS11-12 marine group;Sphingobacteriales NS11-12 marine group | -0,47 | 0,030 | 0,473 |
|  | Campylobacteria;Campylobacterales;Arcobacteraceae;Arcobacter | -0,58 | 0,006 | 0,415 |
|  | Gammaproteobacteria;Alteromonadales;Colwelliaceae;Colwellia | -0,52 | 0,016 | 0,415 |
|  | Bacteroidia;Flavobacteriales;Flavobacteriaceae;Maribacter | 0,50 | 0,020 | 0,425 |
|  | OM190;unknown OM190;unknown OM190;unknown OM190 | -0,45 | 0,039 | 0,504 |
|  | Gammaproteobacteria;Betaproteobacteriales;Methylophilaceae;Methylotenera | 0,43 | 0,049 | 0,571 |
|  | Alphaproteobacteria;Rhodobacterales;Rhodobacteraceae;Sedimentitalea | 0,45 | 0,040 | 0,504 |
|  | Alphaproteobacteria;Rhizobiales;Rhizobiaceae;Ahrensia | 0,53 | 0,014 | 0,415 |
|  | Bacteroidia;Flavobacteriales;Crocinitomicaceae;unknown Crocinitomicaceae | 0,53 | 0,013 | 0,415 |
|  | Gammaproteobacteria;Oceanospirillales;Nitrincolaceae;Amphritea | -0,49 | 0,023 | 0,425 |
|  |  |  |  |  |
| Family | Bacteroidia;Sphingobacteriales;Sphingobacteriales NS11-12 marine group | -0,47 | 0,030 | 0,652 |
|  | Campylobacteria;Campylobacterales;Arcobacteraceae | -0,58 | 0,006 | 0,420 |
|  | Gammaproteobacteria;Alteromonadales;Colwelliaceae | -0,52 | 0,016 | 0,547 |
|  | OM190;unknown OM190;unknown OM190 | -0,45 | 0,039 | 0,652 |
|  | Gammaproteobacteria;Betaproteobacteriales;Methylophilaceae | 0,43 | 0,049 | 0,663 |
|  |  |  |  |  |
| Order | Bacteroidia;Sphingobacteriales | -0,49 | 0,023 | 0,316 |
|  | Campylobacteria;Campylobacterales | -0,58 | 0,006 | 0,263 |
|  | Alphaproteobacteria;Rhizobiales | 0,44 | 0,049 | 0,408 |
|  | OM190;unknown OM190 | -0,45 | 0,039 | 0,408 |
|  | Alphaproteobacteria;Parvibaculales | 0,49 | 0,023 | 0,316 |

# Supplementary figures

**Fig. S1.** Phylogenetic tree based on 18S rRNA gene analysis of the diatom strains using neighbor-joining and Tamura-Nei distance model. Units of branch length indicate substitutions per site.


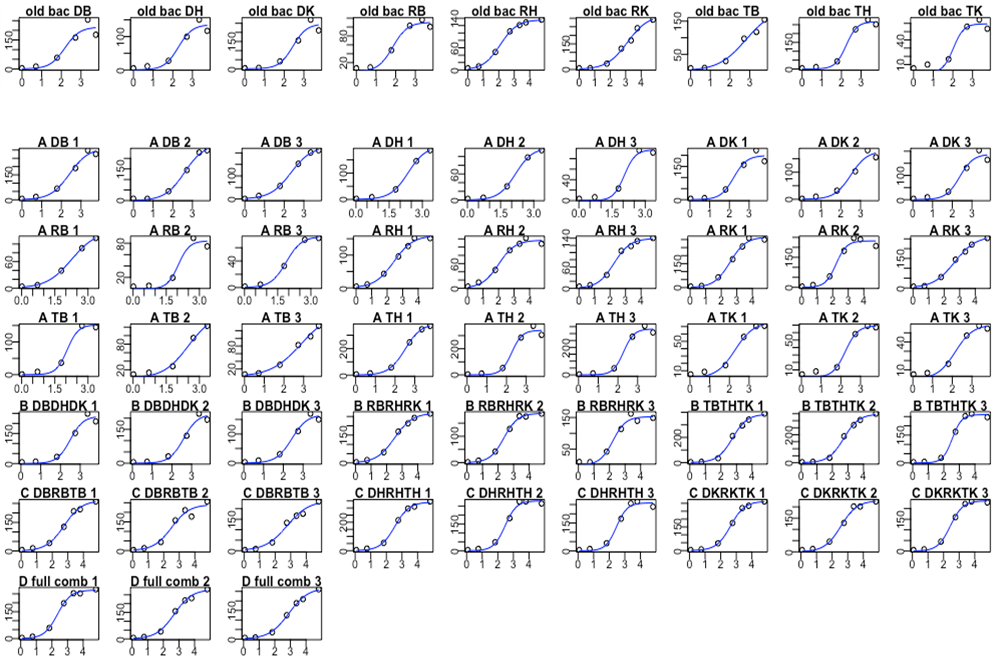


**Fig. S2.** Fitted growth curves based on RFU values over time in days. The first row shows original laboratory cultures, all other rows are inoculated mono- and polycultures. The combinations of species/strains are abbreviated as follows: D = *D. brightwellii*, R = *R. setigera*, T = *T. nitzschioides*. B = Borkum, H = Helgoland, K = Kiel. The number depicts the replicate.

**
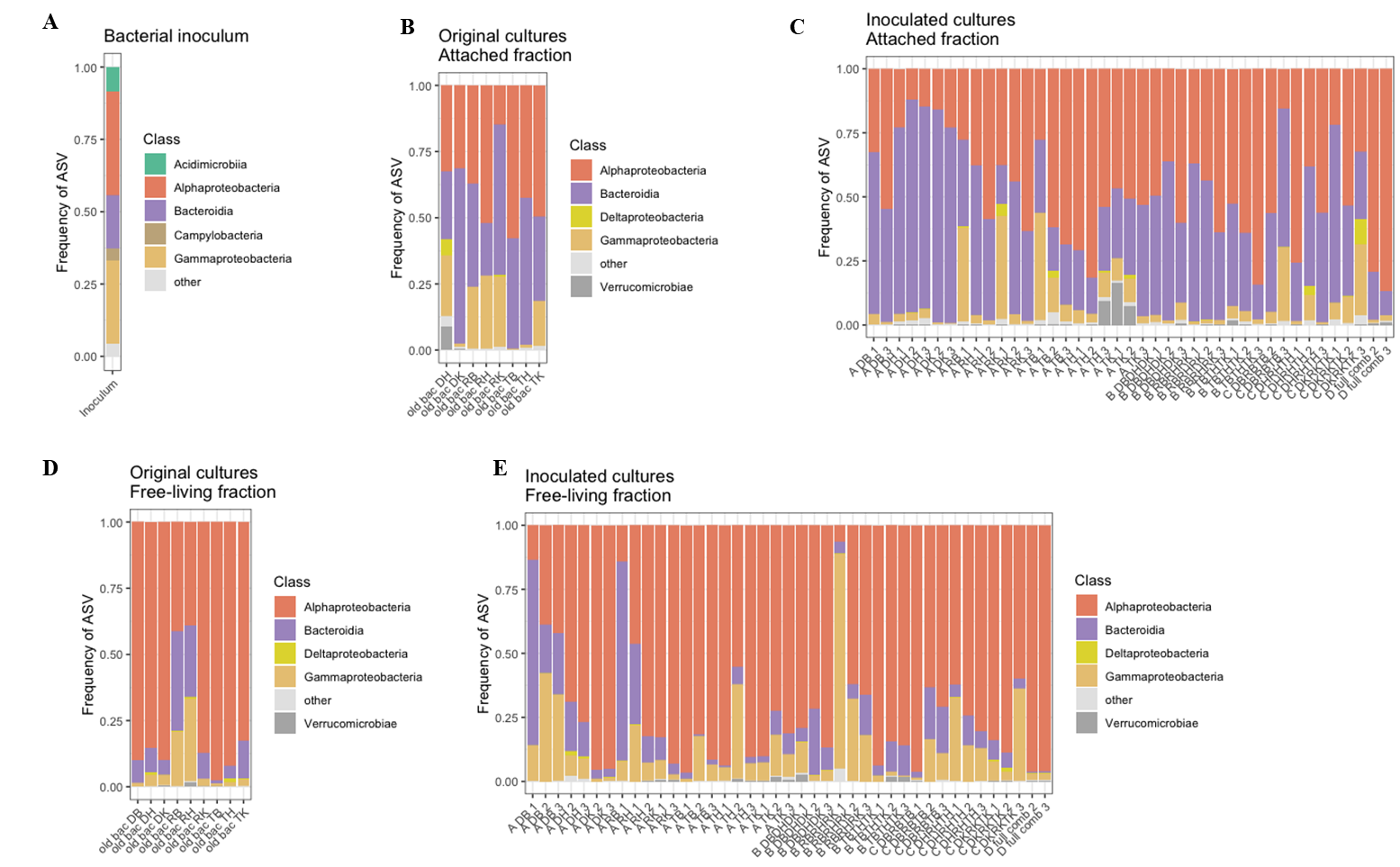
**

**Fig. S3.** Relative abundances of the 5 most abundant bacterial classes. Composition of the bacterial inoculum (A); the attached fraction in original cultures (B) and inoculated cultures including polyculture treatments (C); and the free-living fraction in original cultures (D) and inoculated cultures including polyculture treatments (E). Treatment abbreviations: D = *D. brightwellii*, R = *R. setigera*, T = *T. nitzschioides*. B = Borkum, H = Helgoland, K = Kiel.

**
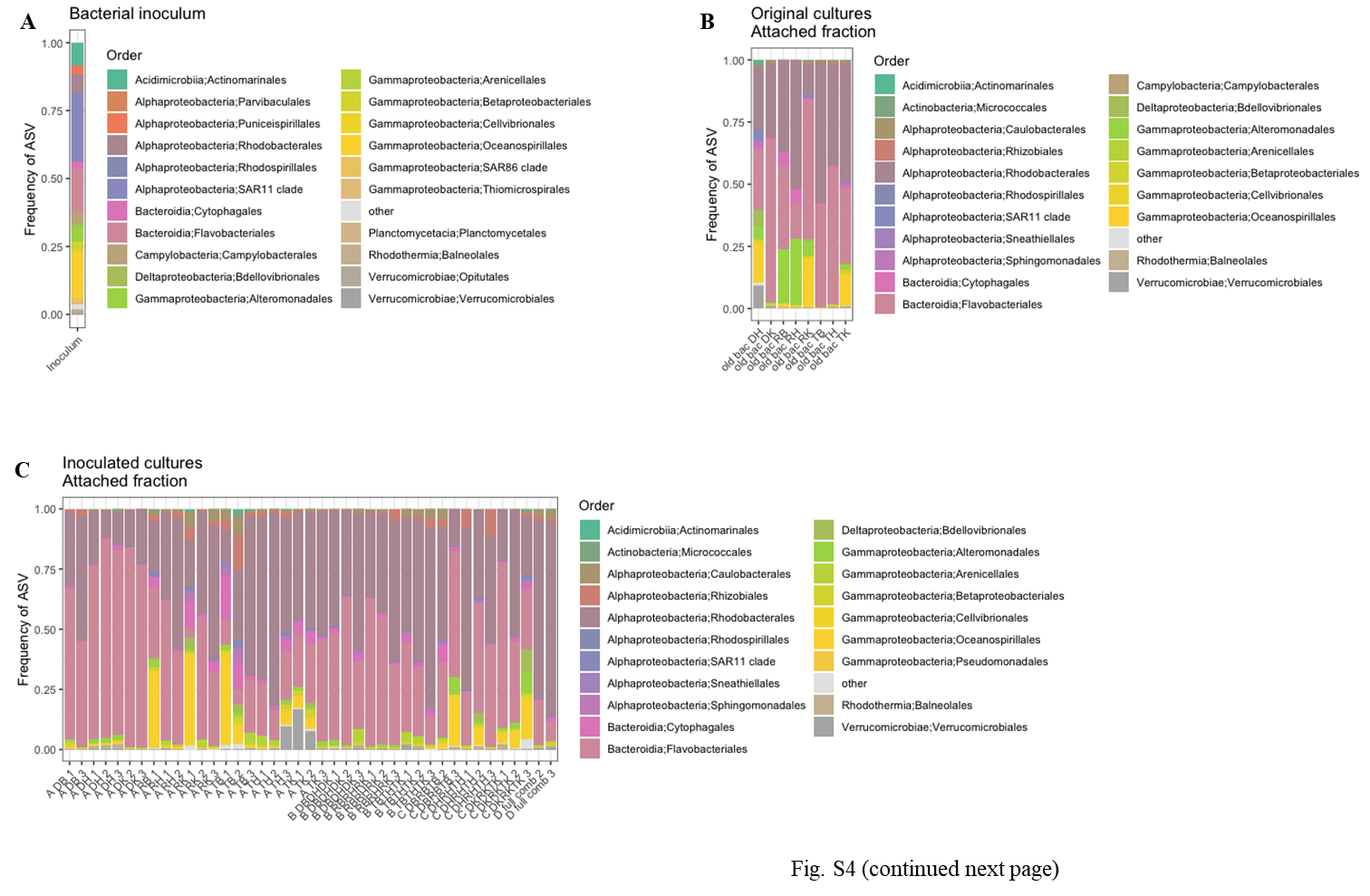
**

**
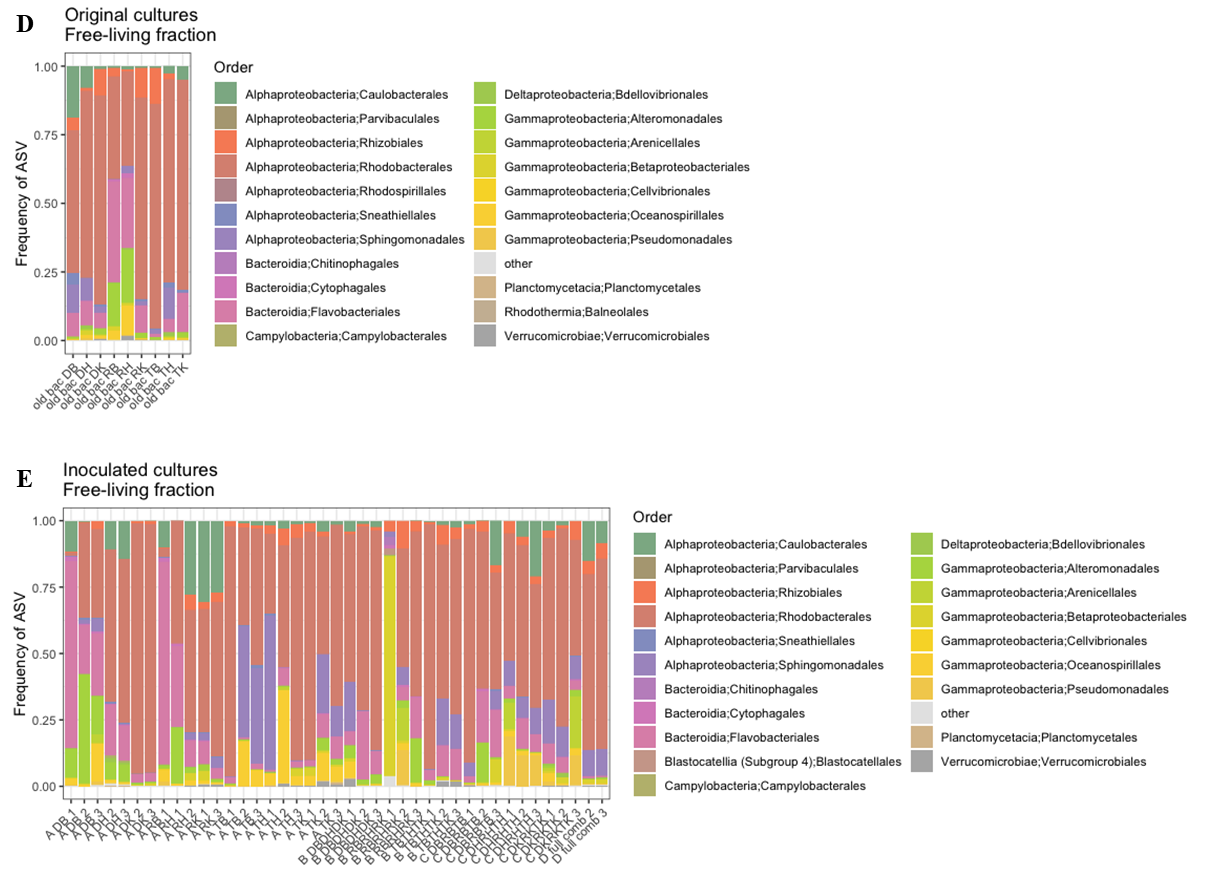
**

**Fig. S4.** Relative abundances of the 20 most abundant bacterial orders. Composition of the bacterial inoculum (A); the attached fraction in original cultures (B) and inoculated cultures including polyculture treatments (C); and the free-living fraction in original cultures (D) and inoculated cultures including polyculture treatments (E). Treatment abbreviations: D = *D. brightwellii*, R = *R. setigera*, T = *T. nitzschioides*. B = Borkum, H = Helgoland, K = Kiel.


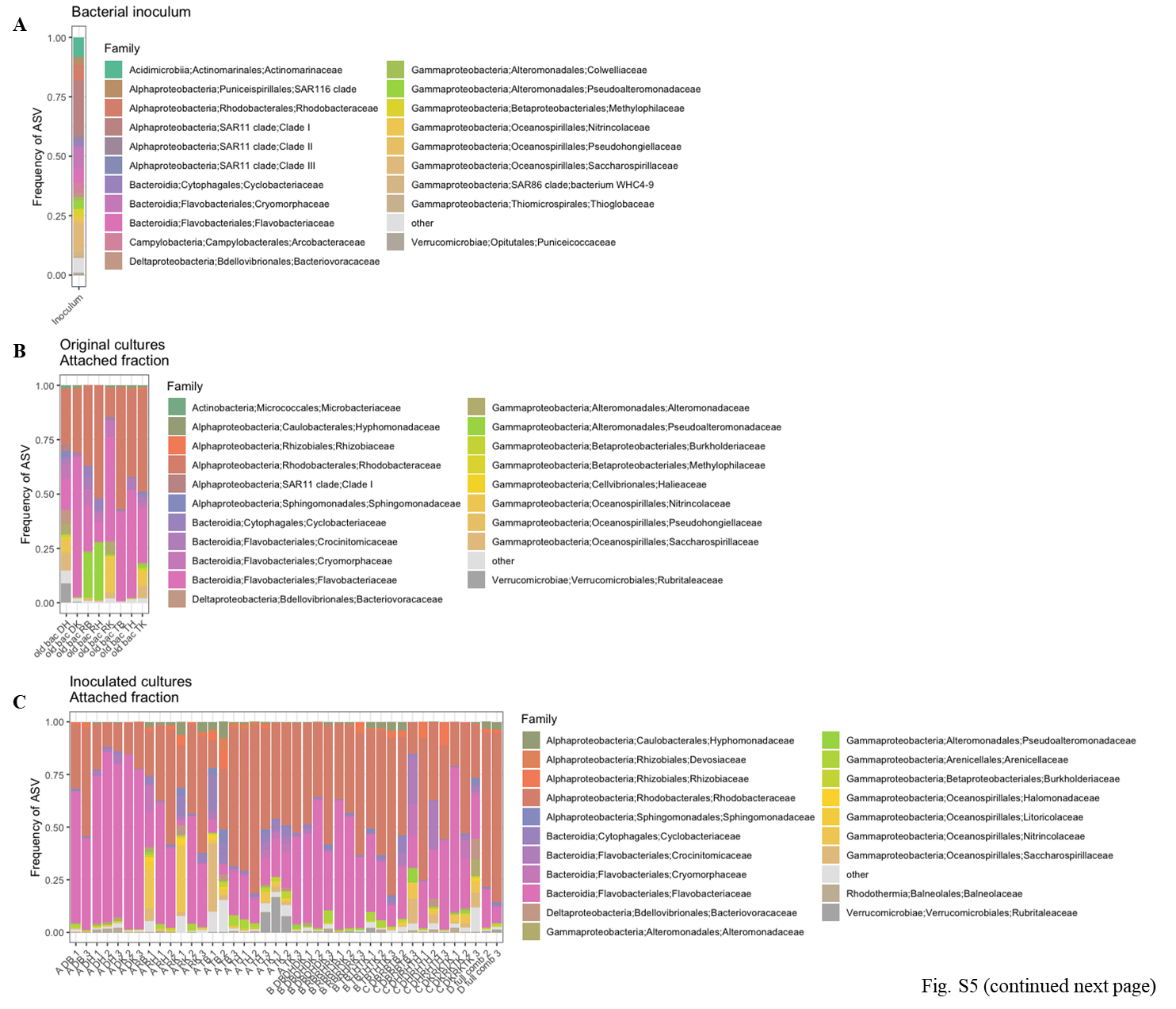


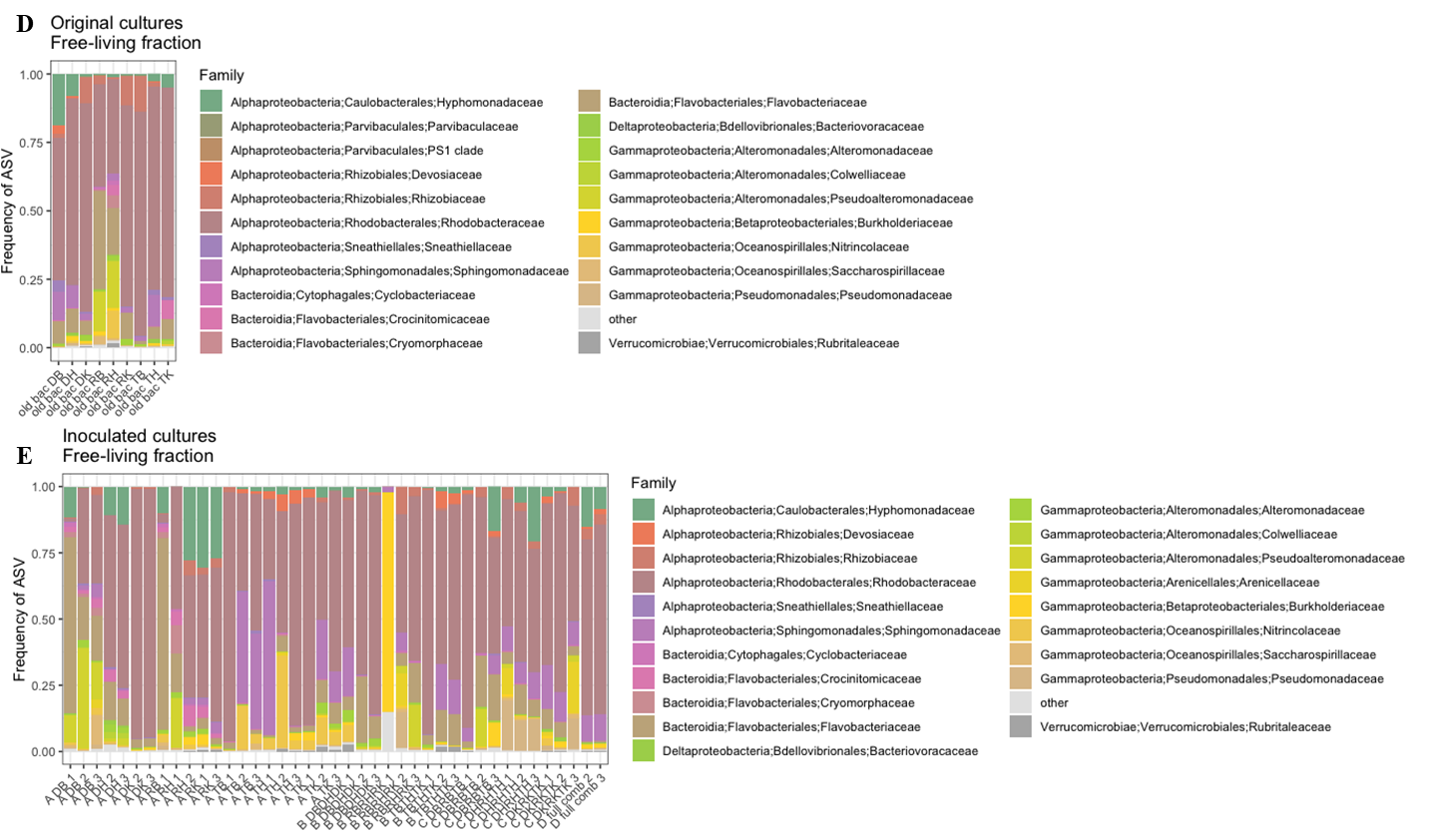


**Fig. S5.** Relative abundances of the 20 most abundant bacterial families. Composition of the bacterial inoculum (A); the attached fraction in original cultures (B) and inoculated cultures including polyculture treatments (C); and the free-living fraction in original cultures (D) and inoculated cultures including polyculture treatments (E). Treatment abbreviations: D = *D. brightwellii*, R = *R. setigera*, T = *T. nitzschioides*. B = Borkum, H = Helgoland, K = Kiel.

**
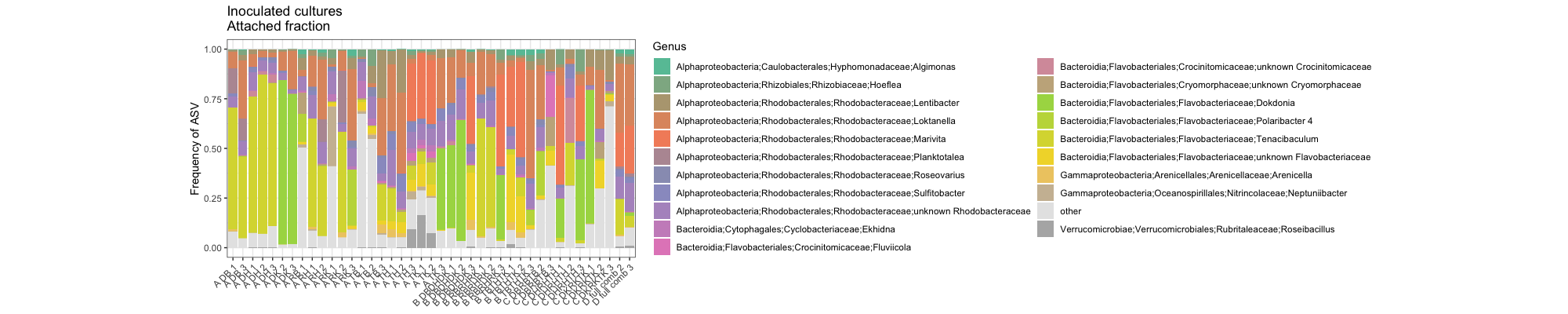
**

**A**


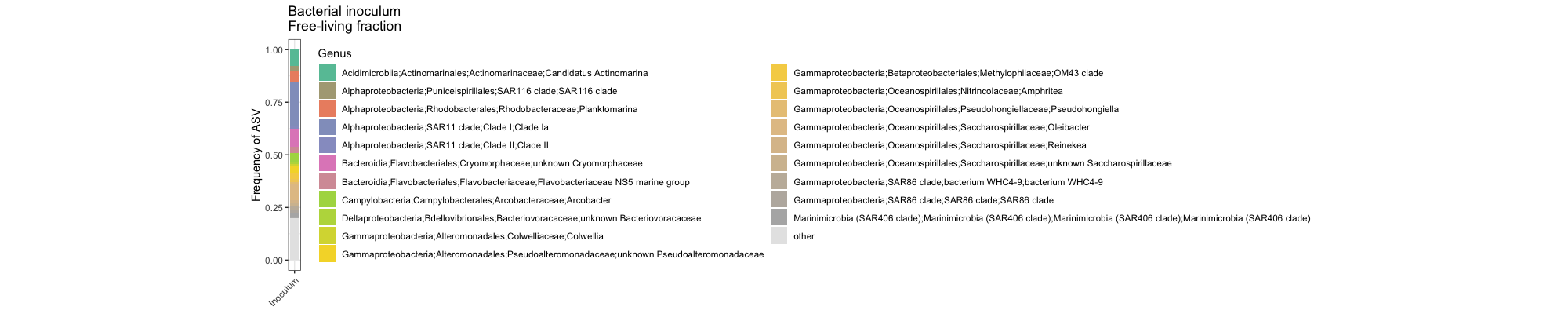

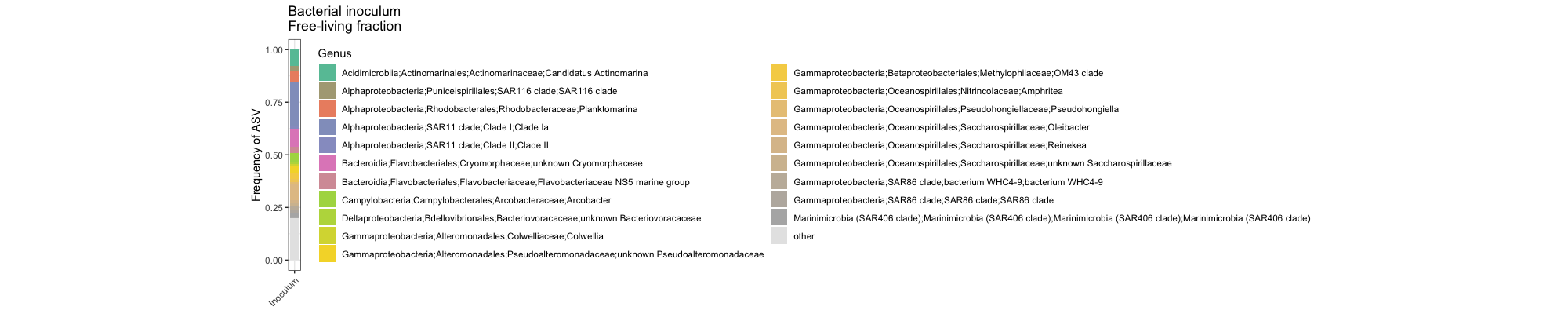


**
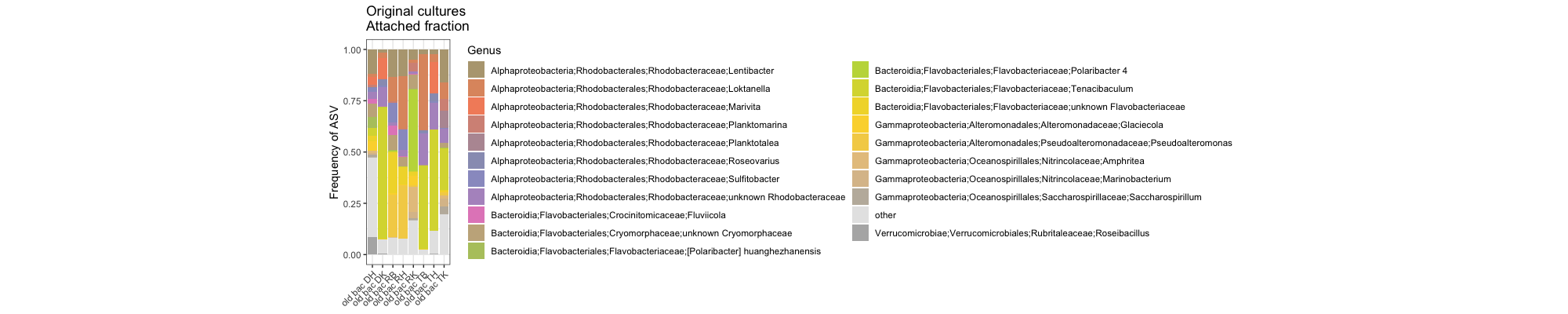
**

**C**

**B**

Fig. S6 (continued next page)

**
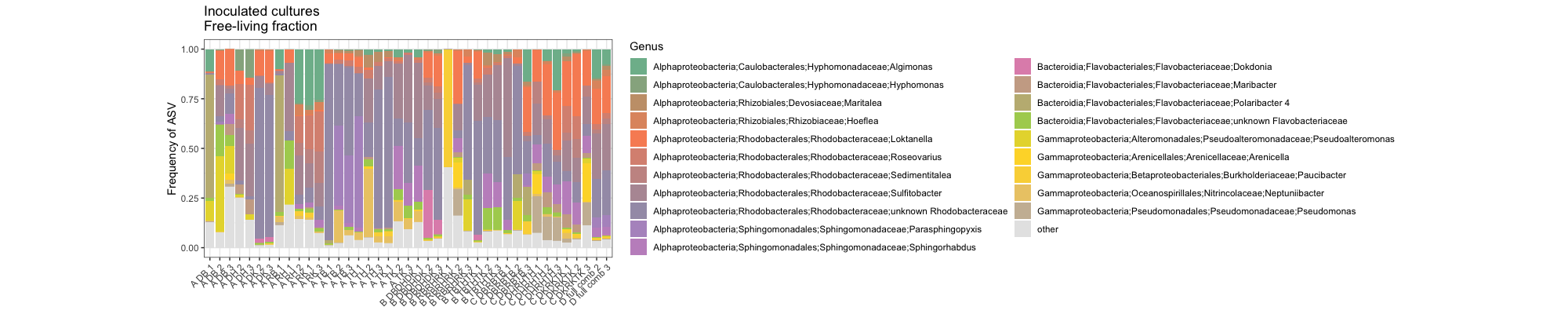

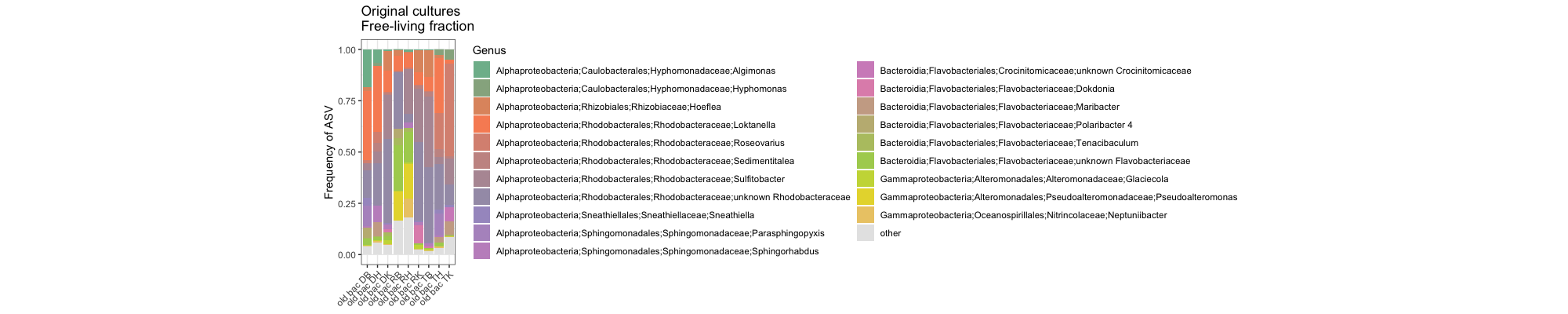
**

**E**

**D**

**Fig. S6.** Relative abundances of the 20 most abundant bacterial genera. Composition of the bacterial inoculum (A); the attached fraction in original cultures (B) and inoculated cultures including polyculture treatments (C); and the free-living fraction in original cultures (D) and inoculated cultures including polyculture treatments (E). Treatment abbreviations: D = *D. brightwellii*, R = *R. setigera*, T = *T. nitzschioides*. B = Borkum, H = Helgoland, K = Kiel.


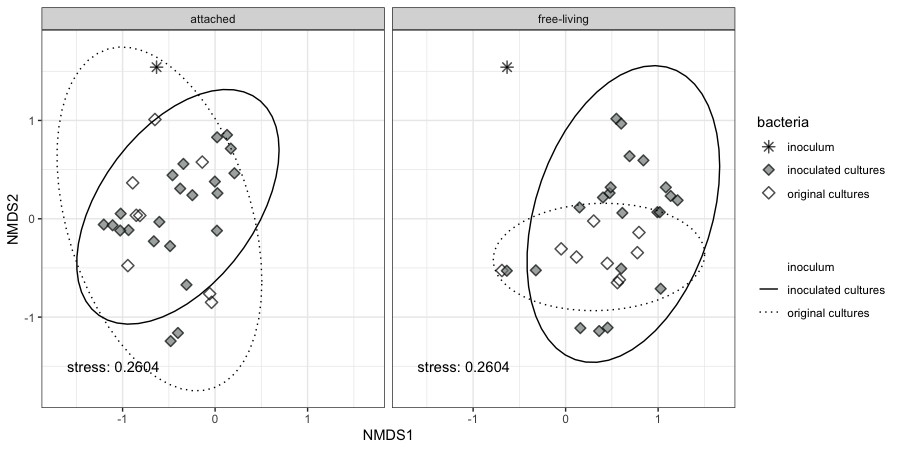


**Fig. S7.** NMDS plots for original and inoculated diatom monocultures for the attached and free-living fraction compared with the bacterial inoculum. Clustering is by inoculation status.


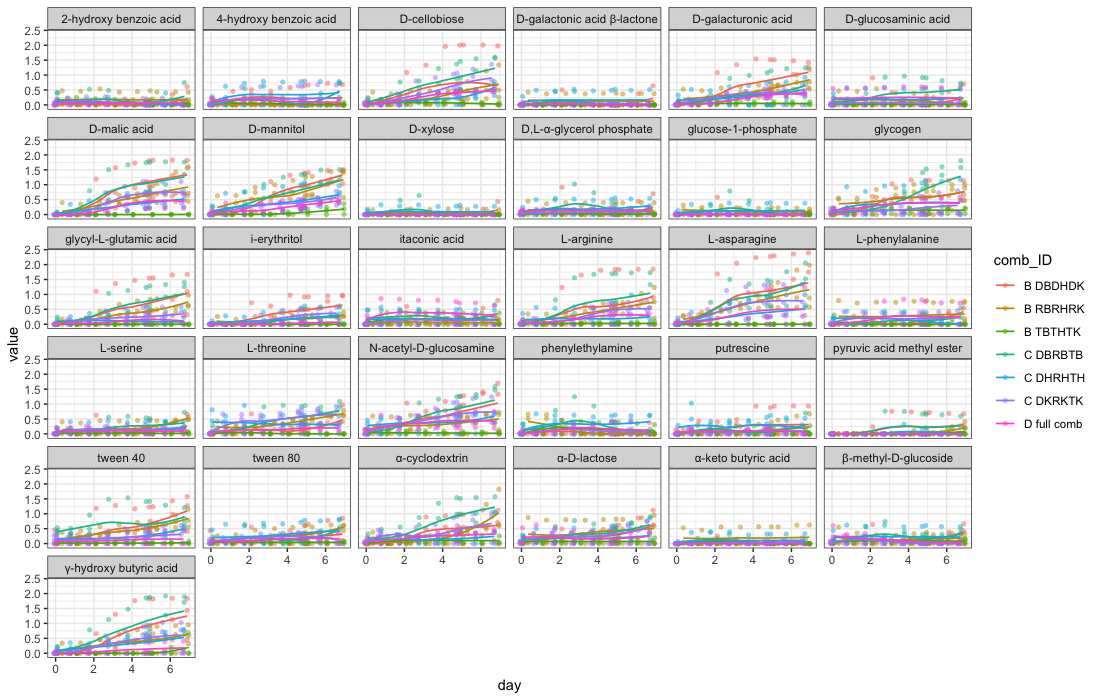


**A**

**B**


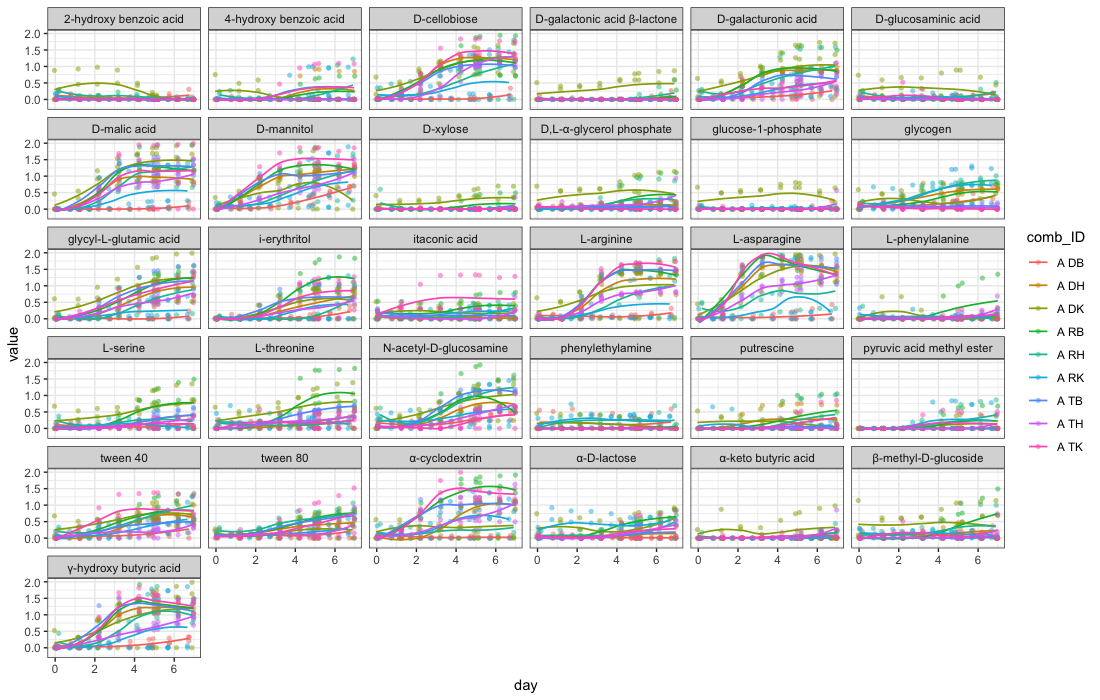


Treatment combination

OD_590_

Treatment combination

OD_590_

**Fig. S8.** Optical density (OD) for single substrates as time series, for diatom monocultures (A) and polycultures (B). Species/origins are abbreviated as: D = *D. brightwellii*, R = *R. setigera*, T = *T. nitzschioides,* B = Borkum, H = Helgoland, K = Kiel.


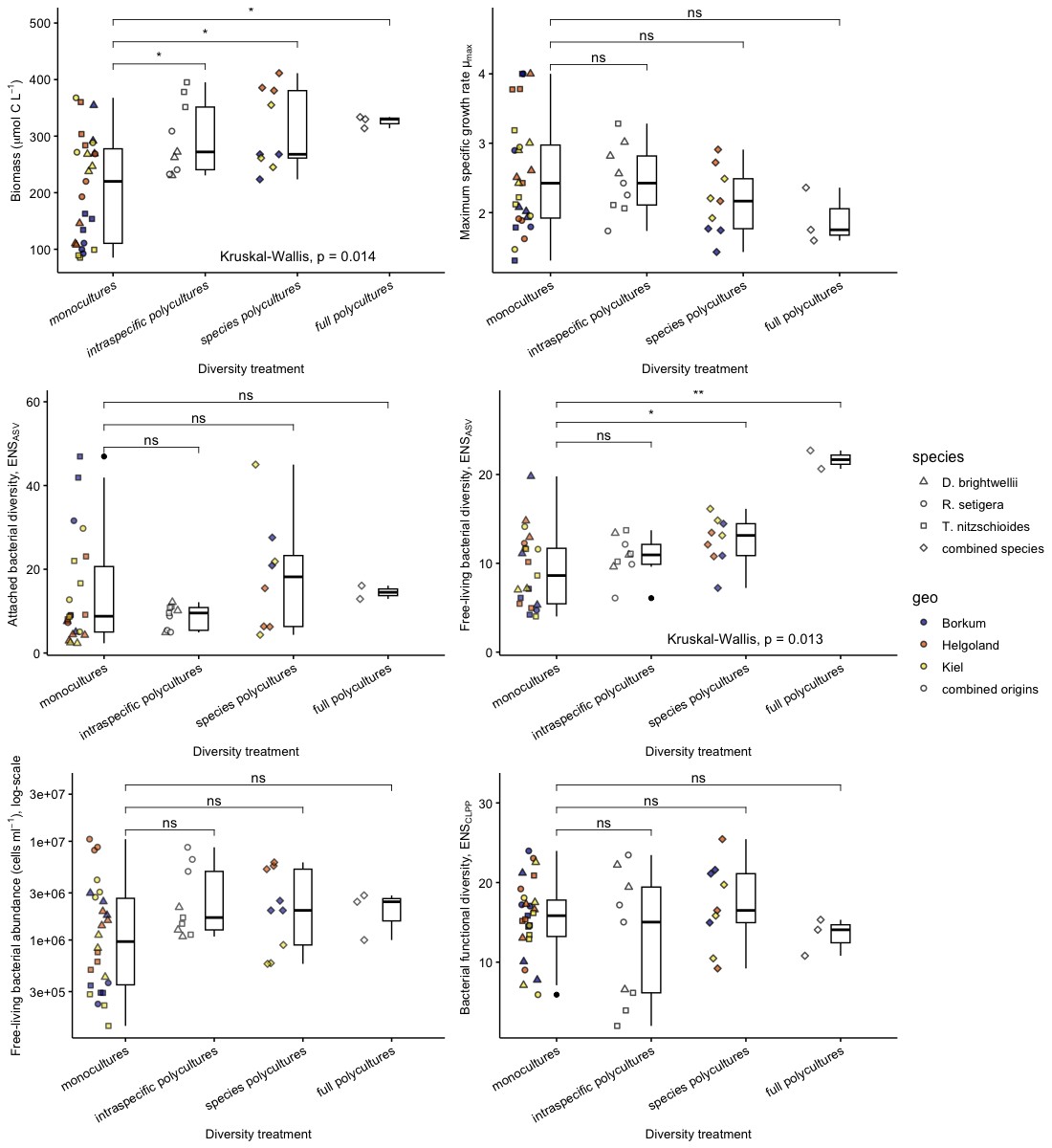


A

B

C

D

E

F

**Fig. S9.** Effects of diatom species and intraspecific richness on phytoplankton community biomass (A), growth rate (B), alpha-diversity ENS_ASV_ of the attached (C) and free-living fractions (D), free-living bacterial abundance (E), and substrate-use diversity ENS_CLPP_ (F).


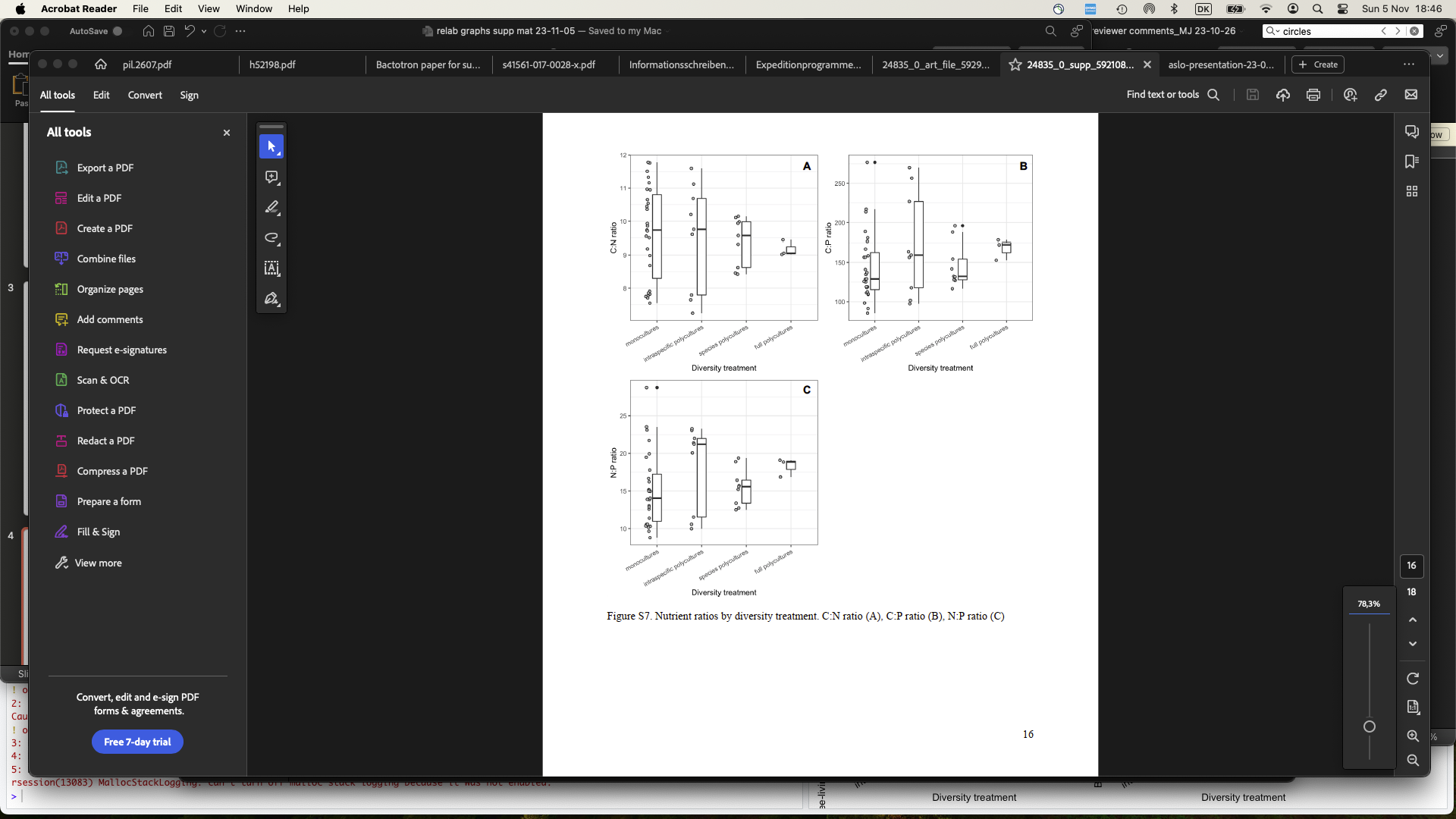


**Fig. S10.** Nutrient ratios by diversity treatment. C:N ratio (A), C:P ratio (B), N:P ratio (C)


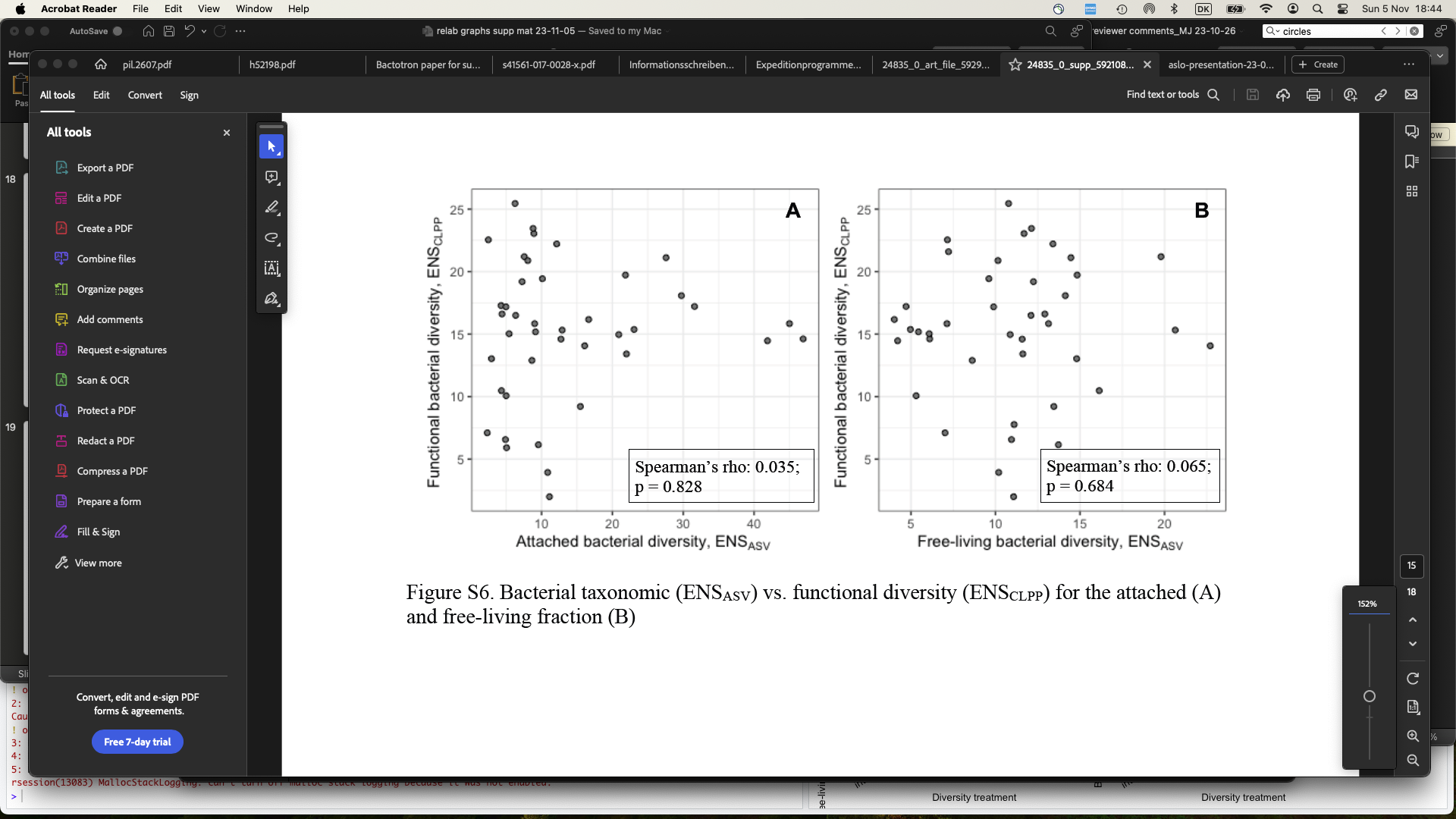


**Fig. S11.** Bacterial taxonomic (ENS_ASV_) vs. functional diversity (ENS_CLPP_) for the attached (A) and free-living fraction (B)


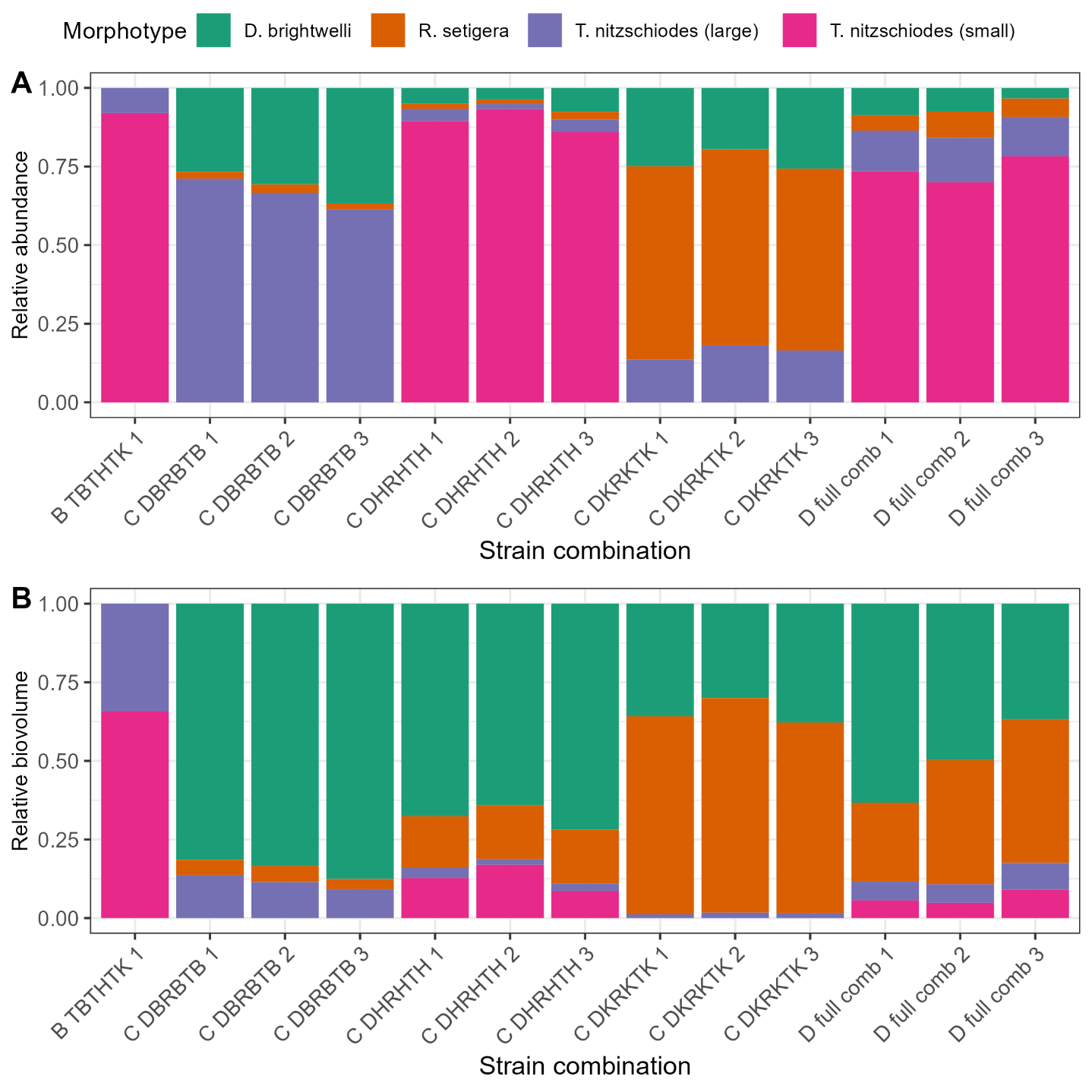


**Fig. S12:** Relative abundances in terms of cell density (A) and relative biovolumes (i.e., a proxy for biomass; panel B) of diatom morphotypes in polycultures containing species mixes. Estimates for the “small” and “large” morphotypes of *T. nitszchiodes* are also shown for this intraspecific polyculture, as the smaller cells in TH were clearly distinguishable from the other two strains.


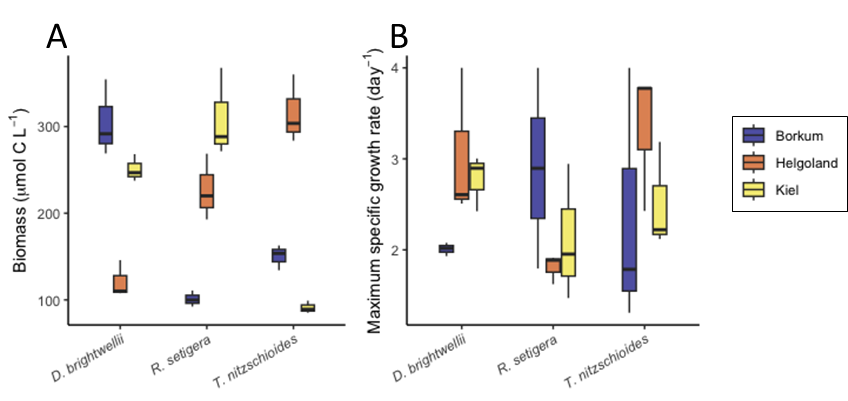


**Fig. S13.** Diatom monoculture biomass (A) and growth rate (B)


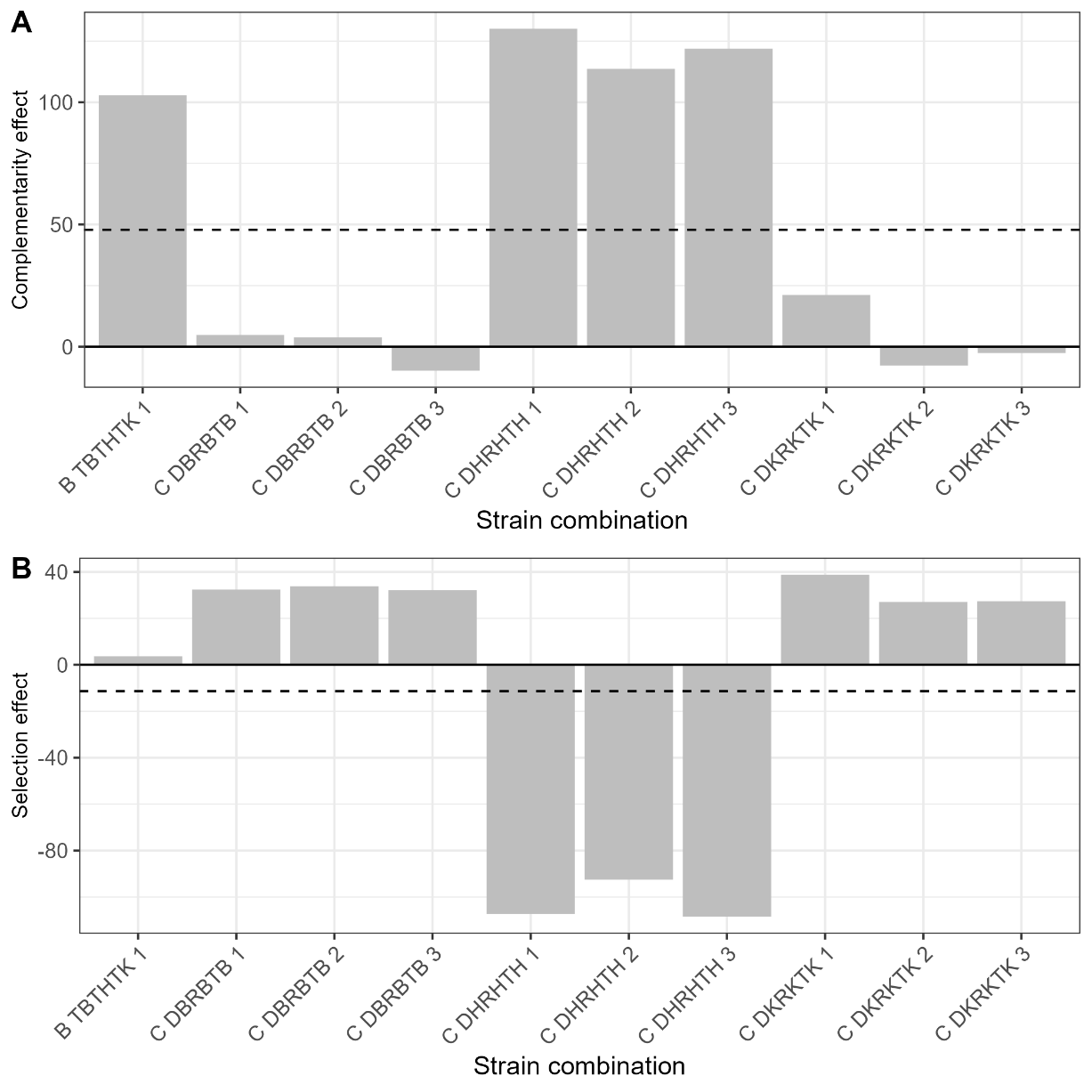


**Fig. S14.** Complementarity effects (A) and selection effects (B) on biomass (units in µmol C/l) from experimental units where these estimates were possible (i.e., estimates of relative biovolumes per visually distinguishable morphotype could be made). The sum of complementarity and selection effects equals the net biodiversity effect (NBE, Fig. 1). These effects were estimated following Loreau and Hector [2] for the species polycultures (i.e., morphologically distinct species) and for one replicate of the *Thalassionema* intraspecific polyculture (i.e., with 2 visually distinct morphotypes). Dashed lines show average effects across the strain combinations shown. These estimates suggest that complementarity effects for these treatments were generally positive (mean 47.8, 95% CI: (4.5, 91.2)) while selection effects were near zero on average (mean -11.3, 95% CI: (-57.1, 34.4)), though effects varied widely by species/strain combination.

# Supplementary methods

**Bacterial substrate use characterization:** EcoPlates™ contain 31 different substrates arranged on well plates (see Table S3 for substrates); we inoculated each well with a 150 µL aliquot from each experimental unit within 12 hours of bacterial sampling and incubated plates in the dark at 18°C. Optical density (OD) was measured photometrically at 590 nm [3] every 24 hours for 7 days. Raw OD values were corrected for OD of seawater. Values above OD 0.1 were considered a positive response. Resulting net OD values on day 7 were chosen for substrate use profiles.

**Elemental composition analysis:** For elemental composition analysis, which we used to assess the role of nutrient use/stoichiometry across diversity treatments, 3 x 50 ml of culture were filtered onto acid-washed precombusted glass-fibre filters (Whatman GF/C). Carbon and nitrogen were measured on an elemental analyzer (ThermoFlash EA1112, Thermo Fisher Scientific Inc., Waltham, Massachusetts, USA). Particulate phosphorus was measured using a molybdate reaction after sulfuric acid digestion [4].

**Processing of DNA samples:** DNA extracts of the free-living fraction were additionally purified to remove residual contaminants using a DNeasy PowerSoil Pro. Kit according to the manufacturer’s instructions (Qiagen, Düsseldorf, Germany). For amplifying target amplicons in polymerase chain reaction (PCR), a 515F-Y (5′-GTGYCAGCMGCCGCGGTAA) primer and a 926R (5′-CCGYCAATTYMTTTRAGTTT) primer covering the V4 and V5 regions of the 16S rRNA gene were used [5]. 10 individual barcodes were used for both forward and reverse primers resulting in 100 possible barcode combinations. Mock communities containing 8 (even community) and 22 (staggered community) clones from 16S and 18S rRNA genes [5] were used as an internal standard to validate sequencing runs. 12.5 μl PCR water was mixed with 10 μl of mastermix containing Taq DNA polymerase and deoxyribose nucleoside triphospates (dNTP) (5 PRIME HotMasterMix, Quantabio, Beverly, Massachusetts, USA). 0.75 μl of the assigned forward and reverse primers and 1 μl (DNA concentration >2 ng/μl) or 2 μl (<2 ng/μl) of DNA were added. PCR was performed with an initial denaturation step (95° C for 102 sec) and 25 cycles of 95° C (45 sec), 50° C (45 sec) and 68° C (90 sec), followed by a final elongation step (68° C for 300 sec). Presence of amplification products was confirmed by gel electrophoresis (Power Pac 300, Bio-Rad, Hercules, California, USA). PCR products were purified using AMPure XP magnetic beads (Beckmann Coulter, Brea, California, USA) and DNA concentrations were adjusted to 1 ng/μl in TE buffer. 1 μl of every sample including the mock communities were pooled for attached and free-living bacterial fractions separately. The pools were purified according to the manufacturer’s instructions using SPRIselect magnetic beads (Beckmann Coulter), eluted with 35 μl TE buffer, and stored at -20° C until sequencing. Pools were sequenced at the Deutsche Sammlung von Mikroorganismen und Zellkulturen (DSMZ, Braunschweig, Germany) on an Illumina Miseq – Short Read Sequencer (San Diego, California, USA) in 2x300 bp mode and demultiplexed at the sequencing site. Bioinformatic processing of the raw sequences was performed according to a pipeline described in [6].

**References**

1. Sala MM, Arrieta JM, Boras JA, Duarte CM, Vaqué D. The impact of ice melting on bacterioplankton in the Arctic Ocean. *Polar Biol* 2010; **33**: 1683–1694.

2. Loreau M, Hector A. Partitioning selection and complementarity in biodiversity experiments. *Nature* 2001; **412**: 72–76.

3. Lehman RM, Colwell FS, Ringelberg DB, White DC. Combined microbial community-level analyses for quality assurance of terrestrial subsurface cores. *J Microbiol Methods* 1995; **22**: 263–281.

4. Grasshoff K, Ehrhardt M, Kremling K, Anderson LG (eds). Methods of seawater analysis, 3rd, completely rev. and extended ed ed. 1999. Wiley-VCH, Weinheim ; New York.

5. Parada AE, Needham DM, Fuhrman JA. Every base matters: assessing small subunit rRNA primers for marine microbiomes with mock communities, time series and global field samples. *Environ Microbiol* 2016; **18**: 1403–1414.

6. Yeh Y-C, Needham DM, Sieradzki ET, Fuhrman JA. Taxon disappearance from microbiome analysis reinforces the value of mock communities as a standard in every sequencing run. *MSystems* 2018; **3**.
